# Ca^2+^ efflux via plasma membrane Ca^2+^-ATPase mediates chemotaxis in ascidian sperm

**DOI:** 10.1038/s41598-018-35013-2

**Published:** 2018-11-09

**Authors:** Kaoru Yoshida, Kogiku Shiba, Ayako Sakamoto, Jumpei Ikenaga, Shigeru Matsunaga, Kazuo Inaba, Manabu Yoshida

**Affiliations:** 1 Faculty of Biomedical Engineering, Toin University of Yokohama, Yokohama, Kanagawa 225-8503 Japan; 2 Misaki Marine Biological Station, School of Science, the University of Tokyo, Miura, Kanagawa 238-0225 Japan; 3 Shimoda Marine Research Center, University of Tsukuba, Shimoda, 415-0025 Japan; 4 Present Address: Division of Structural and Synthetic Biology, RIKEN Center for Life Science Technologies, Yokohama, Kanagawa 230-0045 Japan; 5 Present Address: Central Research Laboratory, Hamamatsu Photonics K.K., Hamamatsu, Shizuoka 434-8601 Japan

## Abstract

When a spermatozoon shows chemotactic behavior, transient [Ca^2+^]_i_ increases in the spermatozoon are induced by an attractant gradient. The [Ca^2+^]_i_ increase triggers a series of stereotypic responses of flagellar waveforms that comprise turning and straight-swimming. However, the molecular mechanism of [Ca^2+^]_i_ modulation controlled by the attractants is not well defined. Here, we examined receptive mechanisms for the sperm attractant, SAAF, in the ascidian, *Ciona intestinalis*, and identified a plasma membrane Ca^2+^-ATPase (PMCA) as a SAAF-binding protein. PMCA is localized in sperm flagella membranes and seems to interact with SAAF through basic amino acids located in the second and third extracellular loops. ATPase activity of PMCA was enhanced by SAAF, and PMCA inhibitors, 5(6)-Carboxyeosin diacetate and Caloxin 2A1, inhibited chemotactic behavior of the sperm. Furthermore, Caloxin 2A1 seemed to inhibit efflux of [Ca^2+^]_i_ in the sperm, and SAAF seemed to competitively reduce the effect of Caloxin 2A1. On the other hand, chemotactic behavior of the sperm was disordered not only at low-Ca^2+^, but also at high-Ca^2+^ conditions. Thus, PMCA is a potent candidate for the SAAF receptor, and direct control of Ca^2+^ efflux via PMCA is a fundamental mechanism to mediate chemotactic behavior in the ascidian spermatozoa.

## Introduction

Ca^2+^ is a well-known second messenger that plays important roles relating to numerous events in virtually all types of cells^1^. Even in spermatozoa, intracellular Ca^2+^ mediates critical events in the fertilization process, namely, activation of sperm motility^2^, sperm chemotaxis^3–5^, capacitation and hyperactivation of motility^6,7^ and the acrosome reaction^8,9^. Especially, Ca^2+^ regulates asymmetric property of flagellar beating^5,10^, and the concentration of intracellular Ca^2+^ is precisely controlled for regulating sperm direction.

The intracellular Ca^2+^ concentration ([Ca^2+^]_i_) is regulated by many Ca^2+^ transport systems in the plasma membrane such as the Ca^2+^ channels contributing to [Ca^2+^]_i_ increase, and, Ca^2+^ exchangers/pumps leading to the [Ca^2+^]_i_ decrease^11,12^. Among them the plasma membrane Ca^2+^-ATPase (PMCA), which belongs to the P-type ATPase family, seems to act as a fine tuner of Ca^2+^ efflux^13^ and maintains Ca^2+^ homeostasis in different kinds of mammalian cells^14^. PMCA also plays a role in modulating the signal transduction pathways. Activity of PMCA seems to be regulated by proteins from the membrane-associated guanylate kinase (MAGUK) family, such as Ca^2+^/calmodulin-activated serine/threonine kinase (CASK)^15,16^. Furthermore, PMCA4 deficiency is known to cause reduced sperm motility in mice^17^.

The chemotactic behavior of a sperm towards an egg plays an important role in ensuring fertilization. It is a widespread phenomenon that occurs in most life forms from lower plants to mammals. This behavior is also mediated by [Ca^2+^]_i_^18^. In the sperm of the ascidian, *Ciona intestinalis*, the sperm-activating and attracting factor (SAAF), which is a sulfate-conjugated hydroxysteroid^19–21^, mediates both activation of sperm motility and chemotaxis^3,19,22^. The phenomena requires extracellular Ca^2+^ ^3,23,24^. To activate the sperm and make it motile, SAAF activates the voltage-dependent Ca^2+^ channel-driven Ca^2+^ influx and the calmodulin/calmodulin kinase II pathway^3,25^. With respect to chemotaxis, the asymmetric waveform of the sperm flagellum, which is important for chemotactic response of the sperm, is mediated by the Ca^2+^ channels^23^. Moreover, we have revealed that transient [Ca^2+^]_i_ increases in the spermatozoon (Ca^2+^ bursts) and is induced in a SAAF gradient, triggering a series of stereotypic responses of flagellar waveforms that comprise turning and straight-swimming^24^.

In contrast to the knowledge on sperm attractants and the participation of Ca^2+^, the sperm receptors for the attractants are almost unknown, and only the attractant receptors of the sea urchin *Arbacia punctulata* and the starfish *Asterias amurensis* have been identified as guanylyl cyclases^26–28^. Therefore, the molecular mechanisms for the [Ca^2+^]_i_ modulation by attractants still remain unknown. In the case of the sea urchin and starfish, cGMP produced by the receptor guanylyl cyclase opens the K^+^-selective cGMP-gated channel, resulting in hyperpolarization^29^. This seems to activate the Na^+^/H^+^ exchanger and leads to alkalization, resulting in Ca^2+^ increase via the alkalization-gated channel CatSper^30^. The involvement of cGMP in regulating sperm function is not known in species other than the echinoderm. In the mammalian sperm, CatSper seems to act as a chemoattractant receptor^31,32^. In the ascidian, cGMP does not seem to be involved in chemotactic behavior^3^, and the SAAF receptor may not be the guanylyl cyclase. Thus, identification of the SAAF receptor is required to understand Ca^2+^ signaling and the molecular mechanisms of ascidian sperm chemotaxis. Furthermore, in the case of any other species, Ca^2+^ influx and [Ca^2+^]_i_ increases in the sperm cell are focused in chemotactic behavior, and Ca^2+^ efflux and [Ca^2+^]_i_ decreases are scarcely examined; despite the need for prompt [Ca^2+^]_i_ decrease, it has been observed only in sperm activation of the sea urchin^33^.

In this study, we attempted to identify the SAAF receptor on the sperm of the ascidian, *C*. *intestinalis*. We show here that the PMCA located on the sperm tail binds to the SAAF and mediates chemotactic behavior in the ascidian. Furthermore, modification of the extracellular Ca^2+^ concentration ([Ca^2+^]_ex_) disrupted the Ca^2+^ bursts and the sperm chemotactic behavior. Thus, PMCA is a potential candidate for the SAAF receptor on the sperm. Regulation of the flagellar waveform in chemotactic behavior requires precise control of the Ca^2+^ efflux.

## Results

### PMCA localized on sperm flagellar membranes acts as a target for SAAF

**Figure 1:**
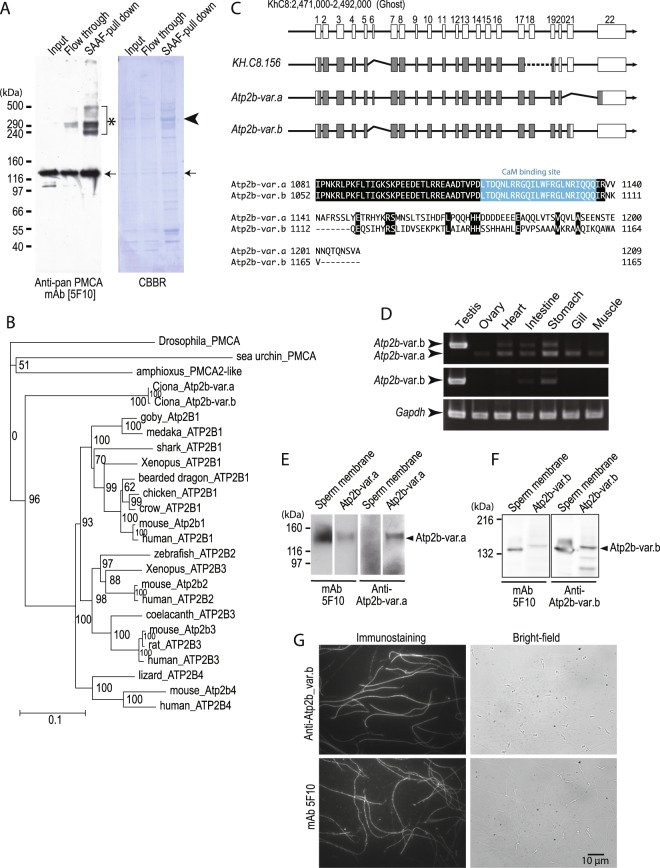
PMCA is the SAAF-binding protein and it is present in spermatozoa. (**A**) Identification of PMCA in the SAAF-binding proteins. Representative results of western blotting using the anti-pan PMCA antibody (5F10) showed a p130 (arrows) and a p370 band (arrowhead). These proteins were identified as PMCA by the PMF method as positive bands between the SAAF-pull down proteins. The aggregation of molecules (asterisk), including p370, was detected as self-aggregations of the p130 PMCA. (**B**) Phylogenetic trees of the PMCA protein family. Drosophila PMCA is used as the outgroup for the PMCA family. Bootstrap values are shown at corresponding branches. (**C**) *Atp2b* gene structure and amino acid sequences. (Upper) A region of the *Atp2b* genome in *C*. *intestinalis* (KhC8:2,471,000-2,492,000) was predicted to have 22 exons. Solid boxes show cording region of genes. In the predicted gene model KH.C8.156, the 18^th^ exon was lost and the 19^th^ exon was changed because of a gap in genome database (shown as a broken line), resulting in error of prediction. (see Supplemental Fig. S3). The two splice variants, *Atp2b*-var.a [LC271262] skips the 21^st^ exon, and *Atp2b*-var.b [LC271263] skips the 6th exon. (Lower) The C-terminus region of the two variants, corresponding to amino acids 1081- of Atp2b-var.a was aligned. The amino acid sequences of the two variants differ from each other just after the CaM-binding domain (blue). (**D**) Expression of the two splice variants in adult tissues. The upper panel shows the RT-PCR reaction using primers set for the 20^th^ and 22^nd^ exons. The middle panel shows the 20^th^ and 21^st^ exons. The bottom panel is the amplification of the household gene glyceraldehyde-3-phosphate dehydrogenase (*Gapdh*). The images were gathered from different gels for juxtaposing. Full-length gels are presented in Supplemental Fig. S4. (**E**,**F**) Expression of the protein *Atp2b*-var.a (**E**) and *Atp2b*-var.b (**F**) in the sperm membrane. Two columns on the left show immunostaining with anti-pan PMCA (5F10). Two columns on the right show immunostaining with anti-*Atp2b*-var.a (**E**) or with anti-*Atp2b*-var.b (**F**) antibodies. In the sperm, only *Atp2b*-var.b was expressed. Predicted molecular weights from deduced amino acid sequences of Atp2b-var.a and Atp2b-var.b are 133 kDa and 128 kDa, respectively. The images of (f) were gathered from different gels for juxtaposing. Full-length gels are presented in Supplemental Figs S5 and S6. Specificity of anti Atp2b-var.b antibody was shown in Supplemental Fig. S7. (**G**) Indirect immunofluorescence assay with the mAb 5F10 and Anti Atp2b-var.b antibody showed that PMCA was selectively localized at the sperm tail (left panels). Scale bar = 10 µm.

In order to identify a receptor for SAAF on the sperm of the ascidian, *C*. *intestinalis*, we attempted to discover SAAF-binding proteins. The SAAF-binding proteins were purified from the sperm membrane fraction by the pull-down assay using a resin conjugated with bio-SAAF, which is the biotinylated derivative of the C(4)-hydroxy group (Supplemental Fig. S1A,B). These were identified by the peptide-mass-fingerprint (PMF) method using MALDI-TOF/MS with the genome database of *C*. *intestinalis* (Ghost: http://ghost.zool.kyoto-u.ac.jp/cgi-bin/gb2/gbrowse/kh/)^34^. The most abundant SAAF-binding protein, the 370-kDa protein (Fig. 1A), was a product of the predicted gene model KH.C8.156, which is similar to the human PMCA3 (plasma membrane Ca^2+^/ATPase 3; ATP2B3) (Supplemental Fig. S1C). Another SAAF-binding protein, the 330-kDa protein was also identified as a product of KH.C8.156 (Supplemental Fig. S1), but the other proteins could not be identified by the PMF method. Thus, we concluded that PMCA is a potent candidate for the SAAF receptor. After searching through the genome database, we only found one PMCA gene (*Atp2b*) in *C*. *intestinalis* (KH.C8.156), which seems to diverge from the common ancestral gene of Atp2b1-4 (Fig. 1B). The SAAF-binding proteins were analyzed by a western blot assay with an anti-pan PMCA antibody (mAb 5F10). PMCA was detected as a 130 kDa band (Fig. 1A, arrow), and, as high molecular weight aggregates in a pull-down fraction by SAAF (Fig. 1A, asterisk), one of which was the 370 kDa protein (Fig. 1A, arrow head). We cloned and sequenced mRNAs of *Atp2b* from the testis cDNA and finally found two splice variants (Fig. 1C, Supplemental Fig. S2). There were differences in usage of the 6^th^ and 21^st^ exons, and, the main difference in the two transcripts was the C-terminus region after the CaM binding site (Fig. 1C). Unexpectedly, both variants (Atp2b-var.a: 133 kDa, Atp2b-var.b: 128 kDa) were different from the predicted product of KH.C8.156 (107 kDa) in the genome database (Fig. 1C). We checked the sequence in the genome database and found that there is a gap in the sequence between exon 17 and 19, resulting in an error of prediction in the product of KH.C8.156 (Supplemental Fig. S3). RT-PCR analysis showed that one of the splice variants (*Atp2b*-var.a) was expressed ubiquitously, and the other one (*Atp2b*-var.b) was highly expressed in the testis and weakly expressed in the stomach, intestine, and heart (Fig. 1D). Furthermore, western blot analysis with variant-specific anti-PMCA antibodies showed that only Atp2b-var.b was present in the sperm plasma membrane (Fig. 1E,F). Immunostaining experiments using the mAb 5F10 revealed that PMCA was present in the sperm flagella (Fig. 1G). Thus, we concluded that Atp2b-var.b is the dominant PMCA isoform in the sperm of *C*. *intestinalis* and is defined as the sperm PMCA.

### SAAF interacts with PMCA and enhances ATPase activity

**Figure 2:**
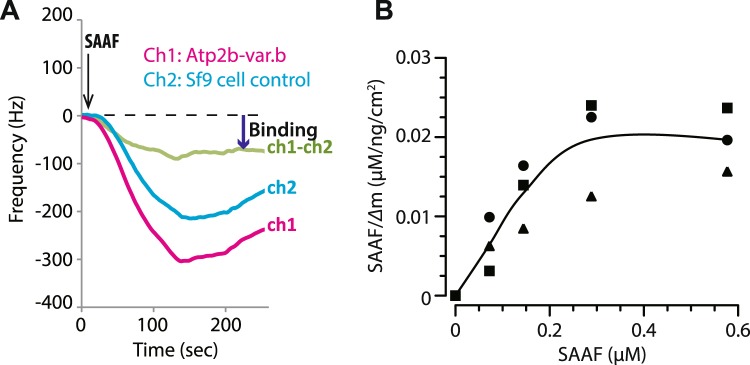
Interaction between SAAF and PMCA. (**A**) Interactions between SAAF and Atp2b-var.b were examined by a highly sensitive 30 MHz quartz crystal microbalance (QCM). The quartz has two channels: wild-type Atp2b-var.b was immobilized on Channel 1 (Ch1: magenta), and the extract of *Sf*9 cells (control) was immobilized on Channel 2 (Ch2: cyan). A decrease in the frequency of the quartz is considered as a mass of molecular binding. (**B**) SAAF binding to wild-type *Ciona intestinalis* PMCA was detected as a differential value (green) of Ch1 and Ch2.

**Figure 3:**
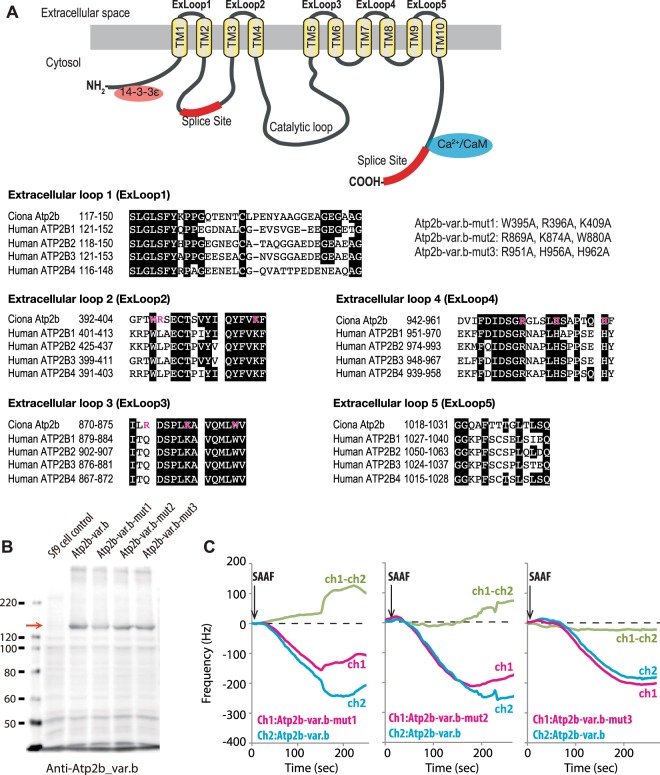
Interaction between SAAF and PMCA mutants. (**A**) Scheme of Atp2b-var.b and the amino acid sequence alignments of the extracellular loops (ExLoops) of PMCAs. The target amino acids for the mutation are shown in magenta. (**B**) Expression of wild-type and mutant Atp2b-var.b proteins in *Sf*9 cells. Western blot results using the anti-Atp2b-var.b antibody showed that Atp2b-var.b proteins were expressed on *Sf*9 cells (arrow). (**C**) Interactions between SAAF and mutant Atp2b-var.b proteins. Atp2b-var.b-mut1 and Atp2b-var.b-mut2 showed reduced interaction, whereas Atp2b-var.b-mut3 showed the same interaction compared to the wild-type.

In order to examine the molecular interaction between PMCA and SAAF, the membrane fraction of *Sf*9 cells expressing recombinant Atp2b-var.b was subjected to an interaction assay with SAAF using a quartz crystal microbalance (QCM) method. PMCA interacts with SAAF and the dissociation constant (K_*D*_) of the interaction between SAAF and Atp2b-var.b was calculated as 240 ± 50 nM (Fig. 2). Next, we attempted to identify the SAAF-binding region in PMCA. The deduced amino acid sequence of Atp2b-var.b was subjected to the TMpred program (http://embnet.vital-it.ch/software/TMPRED_form.html)^35^. Using this program, the membrane-spanning regions and the orientation of the protein were predicted (Fig. 3A, Supplemental Fig. S2). Because SAAF is a sulfate-conjugated polyhydroxysterol, it is highly hydrophilic and negatively charged. The interaction between SAAF and PMCA was proposed to be driven by electrostatic interactions between complementarily charged residues, the positively charged amino acids (basic amino acids) were replaced by neutral amino acids around the extracellular loops (ExLoops) of Atp2b-var.b (Fig. 3A,B). When these mutant-recombinant proteins were compared to the wild-type recombinant proteins, replacing W395A, R396A, and K409A around ExLoop2 (Atp2b-var.b-mut1) and R896A, K874A, and W880A around ExLoop3 (Atp2b-var.b-mut2) reduced the interaction with SAAF (Fig. 3C). On the other hand, replacing R951A, H956A, and H962A around ExLoop4 (Atp2b-var.b-mut3) did not affect the interaction between SAAF and PMCA (Fig. 3C). Thus, PMCA actually binds SAAF directly, and ExLoop2 and ExLoop3 are potent binding sites for SAAF.

**Figure 4:**
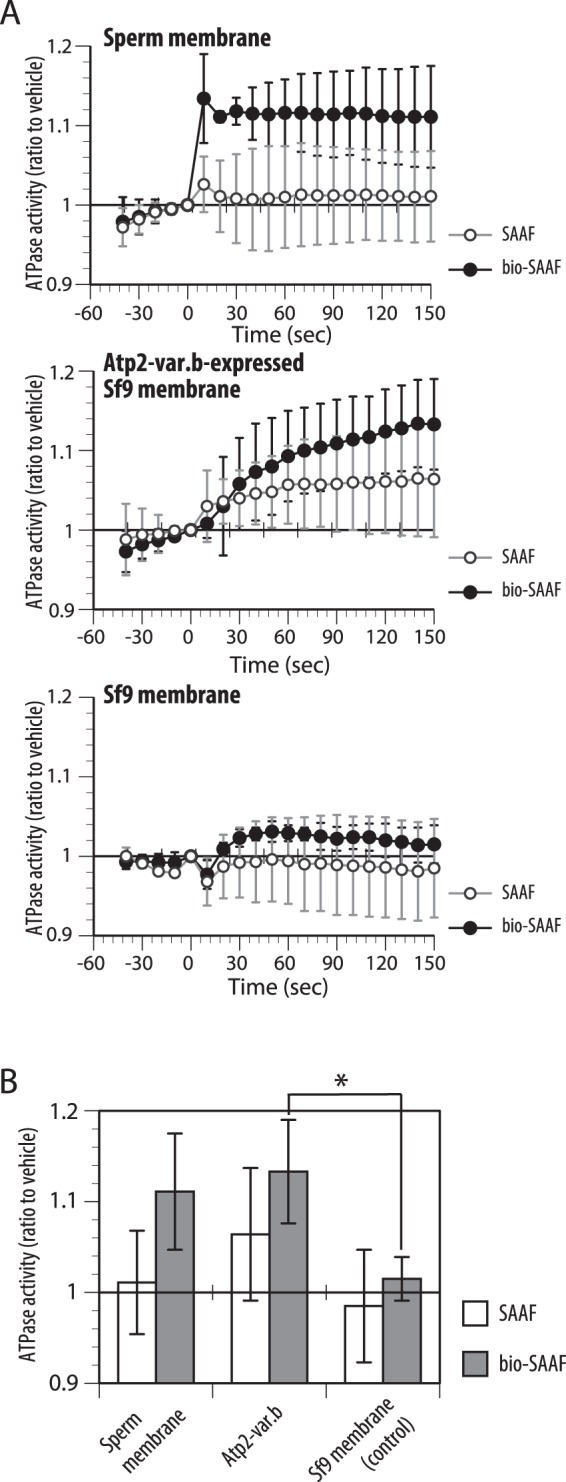
SAAF affects the ATPase activity of PMCA. (**A**) The ATPase activity of the membrane fraction of *C*. *intestinalis*, *Sf*9 cells expressing Atp2b-var.b, and *Sf*9 membranes (control) was measured. Each membrane fraction was pre-incubated with SAAF or with bio-SAAF, and then ATP was added (zero time). Values are expressed as mean ± S.D. of the results from 3 experiments. (**B**) Values at a time of 150 sec are shown. The ATPase activity of the Atp2b-var.b-expressing membrane was significantly activated by bio-SAAF (Student’s t-test: *P < 0.05).

Because PMCA is a P-type primary ion transporter from the ATPase family, we attempted to assay PMCA activity by measuring its ATPase activity. In the assay, membrane fractions of the sperm and of the Atp2b-var.b-expressing *Sf*9 cells were pre-incubated, and then the ATPase activity was measured with and without SAAF and bio-SAAF. Both membranes had activity of an ATPase. However, SAAF increased the activity only in the membrane of Atp2b-var.b-expressing *Sf*9 cells, whereas bio-SAAF increased the activity of both membrane fractions (Fig. 4). Significant increase by bio-SAAF was observed in the membrane of Atp2b-var.b-expressing *Sf*9 cells (Fig. 4B).

### PMCA mediates sperm chemotaxis towards SAAF

**Figure 5:**
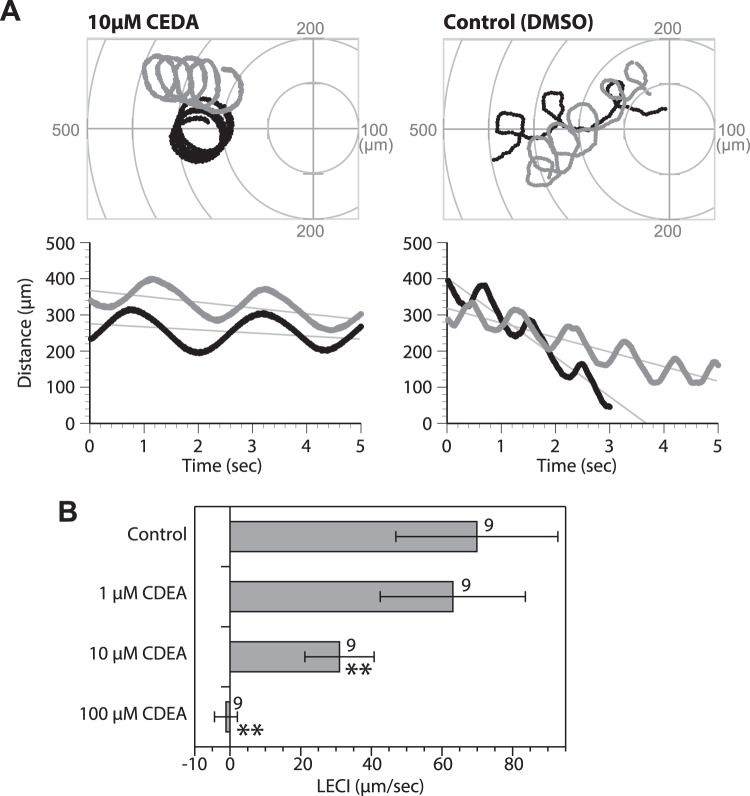
Effect of 5(6)-CEDA on sperm chemotaxis. (**A**) Trajectories and chemotaxis indexes of the sperm near a capillary tube containing 1 µM SAAF. The spermatozoa were treated with 1 µM or 10 µM CEDA succinimidyl ester, or its vehicle (DMSO) for 1.5 h. (Upper) Typical trajectories of two sperm (black and gray lines). The capillary tip was set as the origin of the coordinates. (Lower) Changes in the distance between the capillary tip and the head of the swimming sperm. The lines represent the linear equation of time vs. distance. The chemotaxis index (LECI) is represented by negative values of the coefficients in the equation. (**B**) The average LECI of sperm suspended in artificial sea water (ASW; control) or treated with CEDA. The number shown on the right side of each bar represents number of observed spermatozoa from 3 experiments. Values are expressed as mean ± S.D. CEDA significantly reduced chemotactic behavior of the sperm dose-dependently as compared to the control (Student’s t-test: **P < 0.01).

**Figure 6:**
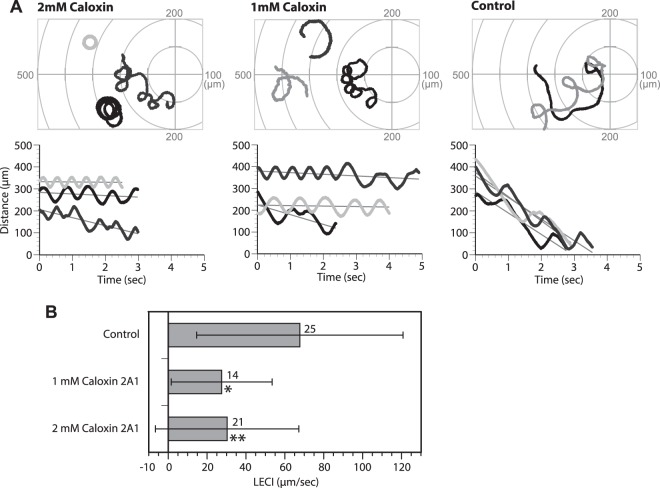
Effect of Caloxin 2A1 on sperm chemotaxis. (**A**) Trajectories and chemotactic indexes of the sperm near a capillary tube containing 1 µM SAAF. The spermatozoa were treated with 1 mM or 2 mM Caloxin 2A1, or ASW for 5 min. (Upper) Typical trajectories of two sperm (black and gray lines). The capillary tip was set as the origin of the coordinates. (Lower) Changes in distance between the capillary tip and the head of the swimming sperm. The lines represent the linear equation of time vs. distance. The chemotaxis index (LECI) is represented by negative values of the coefficients in the equation. (**B**) The average LECI of sperm suspended in artificial sea water (ASW; control) or treated with Caloxin 2A1. The number shown on the right side of each bar represents the number of observed spermatozoa from 7 (2 mM Caloxin), 3 (1 mM Caloxin), and 5 (control) experiments. Values are expressed as mean ± S.D. Caloxin 2A1 significantly reduced chemotactic behavior of the sperm as compared to the control (Student’s t-test: *P < 0.05; **P < 0.01).

SAAF interacts with PMCA and affects its ATPase activity, which could suggest that SAAF modulates Ca^2+^ efflux via PMCA. Thus, we examined the role of PMCA in sperm chemotaxis. When the SAAF capillary was inserted into a sperm solution pre-treated with 5(6)-Carboxyeosin diacetate (CEDA), an inhibitor of PMCA via ATPase activity, most of the sperm lost their chemotactic behavior toward the capillary tip (Fig. 5A). Quantitative analysis of sperm chemotaxis using the linear equation chemotaxis index (LECI)^19^ showed that pre-treatment with 10 µM CEDA partially blocked chemotaxis. The LECI was reduced to one-third that of the control sperm (Fig. 5B). At 100 µM CEDA, sperm chemotaxis was completely blocked and the LECI became almost zero (Fig. 5B). Another PMCA inhibitor Caloxin 2A1, which selectively binds to ExLoop2 of human PMCA^36,37^ also blocked chemotactic response, although, most of sperm was attached to the glass slide (Fig. 6). These results suggest that PMCA contributes to the regulatory mechanism of sperm chemotaxis.

**Figure 7:**
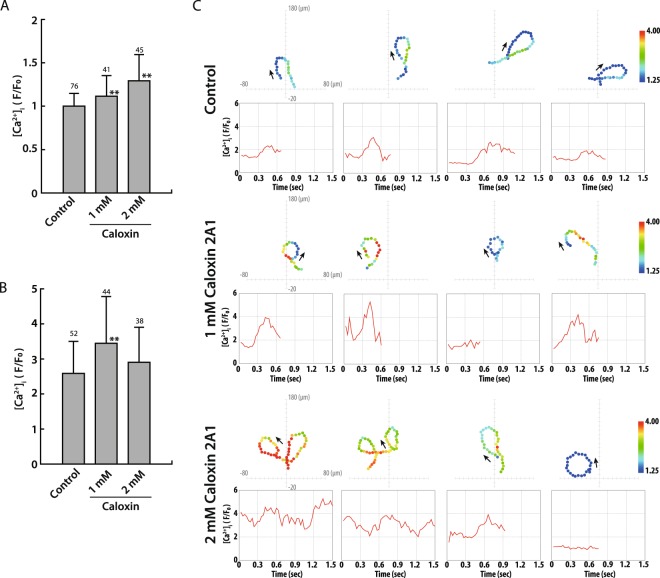
Effects of Caloxin 2A1 on intracellular Ca^2+^ levels and SAAF-induced Ca^2+^ changes. (**A** and **B**) The average of basal [Ca^2+^]_i_ before SAAF stimulation (**A**) or maximum [Ca^2+^]_i_ around the tip of a capillary containing 1 μM SAAF (**B**) in sperm head in ASW (control) or treated with 1 or 2 mM Caloxin 2A1. [Ca^2+^]_i_ was expressed as F/F_0_, which is the value of the fluorescent intensity from head (F) divided by the average intensity of basal [Ca^2+^]_i_ before SAAF stimulation emitted by the heads (F_0_). The values are expressed as mean ± S.D. The number above each bar represents number of observed spermtozoa from 3 experiments. Statistical significance with **P < 0.01 (Student’s t-test) as compared with control. Frequency distribution of [Ca^2+^]_i_ in the sperm is shown in Supplemental Fig. S8B. (**C**) Trajectories of the sperm head (top) and changes in [Ca^2+^]_i_ signals yielded by the sperm head (bottom) in ASW (control) or treated with 1 or 2 mM Caloxin 2A1 around the tip of a capillary containing 1 μM SAAF. The origin of the coordinates indicates the capillary tip. The arrows indicate swimming direction of the sperm. The dots represent not only the head position but also the average intensity of [Ca^2+^]_i_ signals obtained from sperm head in pseudocolors of the LUT. The color scale is the LUT for fluorescence signals.

Caloxin 2A1 inhibited the Ca^2+^ pump of the ascidian PMCA in the absence of SAAF, and Caloxin 2A1 increased the [Ca^2+^]_i_ baseline in the sperm head in a dose dependent manner (Fig. 7A). If the sperm showed a chemotactic response toward SAAF, [Ca^2+^]_i_ during the Ca^2+^ burst should have increased in the presence of Caloxin 2A1 (Fig. 7B,C, and Supplemental Fig. S8). These results suggest that PMCA really reduces the [Ca^2+^]_i_ in the sperm. On the other hand, even in the presence of Caloxin 2A1, the baseline [Ca^2+^]_i_ of some spermatozoa showed a response to SAAF, which was similar to that in the control (Fig. 7C). Even though number of spermatozoa showing higher intensity of [Ca^2+^]_i_ baseline (>1.5) was increased by Caloxin 2A1, 70% (1 mM Caloxin) and 50% (2 mM Caloxin) of the sperm still showed the same [Ca^2+^]_i_ baseline as the control (<1.5) (Supplemental Fig. S8). These results suggest that Caloxin 2A1 inhibits PMCA, and that SAAF activates PMCA competitively with Caloxin 2A1, resulting in maintenance of the [Ca^2+^]_i_ baseline.

### Modification of [Ca^2+^]_ex_ disrupts Ca^2+^ bursts

**Figure 8:**
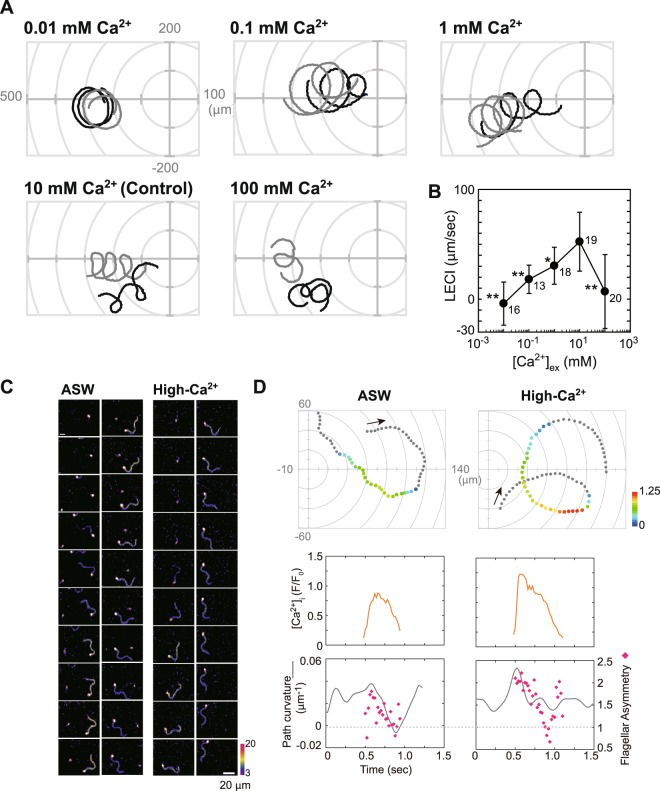
Effects of extracellular Ca^2+^ on the sperm chemotactic behavior and SAAF-induced Ca^2+^ changes. (**A**) Typical trajectories of sperm suspended in low-Ca^2+^ (0.01, 0.1, 1 mM), normal-Ca^2+^ (10 mM, control) or high-Ca^2+^ (100 mM) ASW around the tip of a capillary containing 5 μM SAAF. The origin of the coordinates indicates the capillary tip. (**B**) The LECI of sperm in low and high Ca^2+^-ASW. The LECI is a parameter indicating the strength of sperm-attracting activity. Values are expressed as mean ± S.D. The number above each point represents number of spermatozoa from 3 experiments. Statistical significance with *P < 0.05 and **P < 0.01 (Student’s t-test) as compared to the normal Ca^2+^ ASW. (**C**) Pseudocolor images of [Ca^2+^]_i_ signals yielded from the spermatozoon in ASW or in seawater containing 100 mM Ca^2+^ (high-Ca^2+^) around the tip of a micropipette filled with agar plus 1 µM SAAF at 20 ms intervals. The color scale is the LUT for fluorescence signals. Scale bar = 20 µm. (**D**) Trajectories of the sperm head (top), changes in [Ca^2+^]_i_ signals yielded by the sperm flagellum (middle), and changes in the swimming-path curvature (line) and the asymmetric index (dots) of the spermatozoon (bottom) shown in (**C**). The arrows indicate swimming direction of the sperm. [Ca^2+^]_i_ signals was expressed as F/F_0_, which is the value of the fluorescent signals from flagella (F) divided by the minimum value of signals emitted by the heads (F_0_) as described previously^24^. The origin of the coordinates indicates the micropipette tip. The dots represent not only the head position, but also the average intensity of [Ca^2+^]_i_ signals obtained along the flagellum in pseudocolors of the LUT.

Principal role of PMCA in many mammalian cells is maintenance of Ca^2+^ homeostasis^14^. In the ascidian sperm, inhibition of PMCA resulted in an increase in the [Ca^2+^]_i_ level (Fig. 7). Chemotactic behavior of the spermatozoa as is mediated by Ca^2+^ bursts in the sperm, and the Ca^2+^ bursts require extracellular Ca^2+^ ^24^. Therefore, we examined the effects of the [Ca^2+^]_ex_ on chemotactic behavior. When the [Ca^2+^]_ex_ was lower than 10 mM, the chemotactic response of sperm was concentration-dependent (Fig. 8A,B). At low [Ca^2+^]_ex_ (0.1 mM), changes in the [Ca^2+^]_i_ could not be observed. Interestingly, the chemotactic behavior also decreased when the concentration of [Ca^2+^]_ex_ was higher than 10 mM (Fig. 8A,B). Spermatozoa still demonstrated a chemotactic behavior like ‘turning’ at high [Ca^2+^]_ex_ (100 mM), but the timing of the response was disrupted (Fig. 8C,D). The ‘turning’ response is always observed at the initiation point of the Ca^2+^ bursts^24^. In normal conditions (10 mM [Ca^2+^]_ex_), the Ca^2+^ bursts occur at the Distal Phase^24^ (Supplemental Fig. S9B). However, in the higher [Ca^2+^]_ex_ condition, initiation points of the Ca^2+^ bursts become diverse. The Ca^2+^ bursts are observed even in the Proximal Phase (Supplemental Fig. S9C). Duration of the Ca^2+^ bursts and the [Ca^2+^]_i_ peak of the flagellum at high [Ca^2+^]_ex_ increased as compared to those in the 10 mM [Ca^2+^]_ex_ condition (Supplemental Table S1). Moreover, a decreasing rate of [Ca^2+^]_i_ in the flagella was suppressed at high [Ca^2+^]_ex_ conditions (Supplemental Table S1). Thus, high [Ca^2+^]_ex_ disrupts timing of the Ca^2+^ bursts and potentiates them, resulting in a disruption of the chemotactic response of the sperm flagella. Pattern of the sperm trajectories in the high [Ca^2+^]_ex_ condition (Fig. 8A) does not seem the same as that in the presence of the PMCA inhibitors (Figs 5 and 6). These results support the finding that the [Ca^2+^]_i_ increase during the chemotactic response is a passive phenomenon mediated by some Ca^2+^ channels.

## Discussion

In this study, we revealed that the sperm attractant seems to modulate Ca^2+^ efflux via PMCA on the sperm plasma membrane in the ascidian, *C*. *intestinalis*, resulting in a chemotactic response. Thus, PMCA seems to act as a receptor for SAAF. There are four PMCA genes (*Atp2B1 – 4*) with many splice variants in mammalian and probably in most vertebrate species (see Fig. 1B). Furthermore, PMCA is known as a housekeeping protein for maintaining [Ca^2+^]_i_^38^. We show here that there is only one PMCA gene in the ascidian, *C*. *intestinalis*, and that one of the splice variants, *Atp2b*-*var*.*b*, is specifically expressed on the sperm flagella. Even in mammals, some splice variants of Atp2B2 and Atp2B3 show restricted tissue distribution and seem to be involved in tissue-specific functions^39^. Moreover, PMCA4-deficient male mice are infertile due to a deficiency in sperm motility^17^. Thus, PMCA may be an important signaling molecule in the sperm of many animals.

In this study, we show that SAAF binds to PMCA and accelerates its ATPase activity. Furthermore, changing several amino acids around the extracellular loops 2 and 3 of PMCA result in a loss of SAAF binding. Therefore, the target molecule for SAAF is PMCA, and SAAF seems to induce Ca^2+^ efflux. Interestingly, the ATPase activity of the recombinant PMCA was increased by both SAAF and bio-SAAF, but that of the sperm membrane was activated only by bio-SAAF. Previously we showed that bio-SAAF seemed to have a higher affinity to the sperm than SAAF^40^. Thus, bio-SAAF may bind and activate PMCA constitutively, and that is one reason why bio-SAAF worked as a bait for PMCA in this study. Because of its high affinity, bio-SAAF has no ability for sperm attraction^40^. Since sperm showing chemotactic response should be finely and locally controlled under various SAAF concentrations, the affinity between SAAF and PMCA should not be so high to sense the right concentration. We discuss the affinity between SAAF and PMCA below.

We showed here that two PMCA inhibitors, CEDA and Caloxin 2A1 suppress chemotactic behavior of the sperm, even though their effects differ. CEDA showed stronger inhibitory effects on the chemotactic response than Caloxin 2A1. Generally, PMCA has 10 transmembrane regions and 5 extracellular loops^13^. CEDA probably interacts with the ATP binding region at the intracellular loop between transmembrane 4 and 5, which is identical between the human PMCAs and the *Ciona* PMCA (see Supplemental Fig. S2), and inhibits ATPase activity^37,41^. Thus, effects of CEDA may inhibit other ATPases such as Na^+^/K^+^ pump. On the other hand, Caloxins were developed as the selective peptide inhibitors for human PMCAs as they affect the PMCA activity extracellularly^36^. Caloxin 2A1 selectively binds to ExLoop2 of human PMCA^36,37^ which is conserved between the human PMCAs and the *Ciona* PMCA (see Fig. 3A). As shown in the results, SAAF also binds to ExLoop2; thus, Caloxin 2A1 probably interacts with PMCA and competitively with SAAF. Since affinity of Caloxin 2A1 towards PMCA is relatively low (inhibition constant is 529 µM)^36,37^, Caloxin 2A1 could not inhibit SAAF completely. SAAF kept the [Ca^2+^]_i_ baseline of the sperm same as that of the control even in the presence of Caloxin 2A1 (Supplemental Fig. S8). Interestingly, the pattern of sperm trajectories in the presence of both PMCA inhibitors (Figs 5 and 6) does not seem to be the same as that in the high Ca^2+^ conditions (Fig. 8A). These results show that PMCA is involved in chemotactic behavior of the *Ciona* sperm.

The receptor of sperm attractants has been described in echinoderm species. Specifically, the receptor for sperm attractants in the sea urchin is a transmembrane-type guanylyl cyclase^26^ or a guanylyl cyclase-associated protein^42^. Furthermore, in the starfish, *Asterias amurensis*, it is also a guanylyl cyclase that acts like a receptor^27,28^. In these species, a guanylyl cyclase is activated by the sperm attractant and synthesizes cGMP, resulting in hyperpolarization^29,43,44^ and alkalization of cytosol^30,45^. Finally, alkalization may open the sperm-specific Ca^2+^ channel CatSper, resulting in a [Ca^2+^]_i_ increase^30^. In human sperm, progesterone, one of the potent candidates of the mammalian sperm attractant, seems to bind to the orphan enzyme α/β hydrolase domain–containing protein 2 (ABHD2) and mediates activity of CatSper by depletion of 2-arachidonoylglycerol^46^. Thus, the Ca^2+^ channel CatSper seems to be linked with the receptor of the sperm attractants, and the [Ca^2+^]_i_ increase which is induced by the attractants controls chemotactic behavior of the sperm in these species. On the other hand, the present study shows that PMCA acts as the attractant receptor, and the Ca^2+^ efflux induced by the attractant, mediates sperm chemotaxis in the ascidian. These models are contradictory, but our ascidian model fits our previous results. Addition of SAAF does not induce Ca^2+^ increase, and *Ciona* sperm seems to sense decreases in SAAF concentration, thus resulting in Ca^2+^ bursts^24,47^.

In the present study, we show that normal Ca^2+^ bursts and chemotactic responses were observed only in the presence of 10 mM Ca^2+^. At low [Ca^2+^]_ex_, no [Ca^2+^]_i_ change was observed and the chemotactic response was reduced. At high [Ca^2+^]_ex_, the [Ca^2+^]_i_ increases were potentiated and the chemotactic response was disrupted (Fig. 8). This suggests that the [Ca^2+^]_i_ increase is a passive phenomenon, and that the ascidian tunes its mechanism for controlling sperm flagellar beating in to the seawater condition, which contains 10 mM Ca^2+^.

To examine the molecular mechanisms of sperm attraction, evaluating the binding affinity between the sperm attractant and its receptor is important. In the sea urchin, the binding affinity of the sperm attractant and its receptor seems to be very high. The K_*D*_ was 0.19–15 pM in *Lytechinus pictus*^48^, and 90 ± 84 pM in *Arbacia punctulata*^49^. These K_*D*_ values are almost similar to those of an antibody and its target molecule, that is, the sperm attractant of the sea urchin irreversibly binds to its receptor. In this case, the attractant may affect sperm movement when it binds to the sperm. In fact, guanylyl cyclase, the sperm attractant receptor of the sea urchin, *A*. *punctulata*, is highly dense on the sperm flagellum^49^, and it activates cGMP production and Ca^2+^ fluctuations when it binds to the attractant^50^. On the other hand, in the ascidian, *C*. *intestinalis*, the K_*D*_ value of interaction between the sperm attractant SAAF and PMCA was calculated in the present study and was found to be 240 ± 50 nM. This value is similar to the one calculated for calcium and its chelator or sensor, calmodulin (500 to 5000 nM)^51^, BAPTA (160 to 700 nM)^52^, fluo-4 (345 nM)^53^, etc. We previously estimated that the SAAF gradient around the capillary containing 1 µM SAAF ranges from 10 to 200 nM^24^. That is, the affinity between SAAF and PMCA is suitable for sensing the concentration of the sperm attractant SAAF. Probably, echinoderms and ascidians are using a completely different basic molecular design for sperm chemotaxis even though the regulation of flagellar beating by [Ca^2+^]_i_ is the same. These different results may provide new insights into the specificity and diversity of fertilization mechanisms.

**Figure 9:**
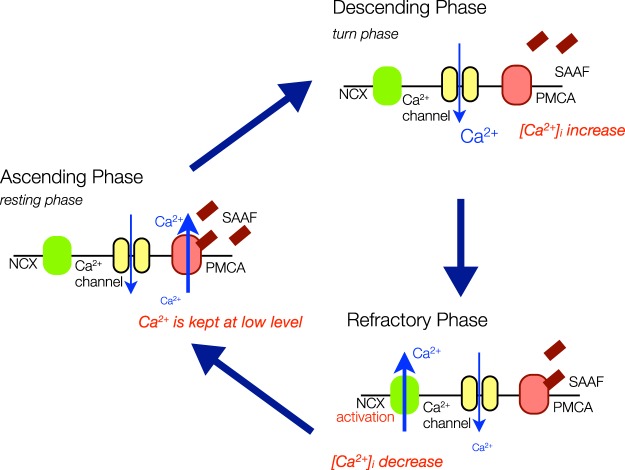
Working hypothesis of the function of SAAF on the sperm plasma membrane. When SAAF concentration increases, PMCA may be continuously activated by SAAF, and Ca^2+^ may be kept at low levels, though Ca^2+^ may enter through Ca^2+^ channel (Ascending Phase). When SAAF concentration decreases, SAAF may detach from PMCA and become inactivated, resulting in [Ca^2+^]_i_ increase (descending phase). Intracellular Ca^2+^ is excreted by NCX and PMCA and finally [Ca^2+^]_i_ reaches low levels (refractory phase). Pair of yellow ovals show Ca^2+^ channel, Red oval: PMCA, Green oval: NCX, Red rectangle: SAAF. Blue arrows indicate flow of Ca^2+^.

We previously demonstrated that NCX, another molecule responsible for Ca^2+^ efflux on the plasma membrane, exists on the ascidian sperm flagella and contributes towards the control of transient [Ca^2+^]_i_ in chemotactic behavior. Inhibition of NCX decreased the alteration of swimming-path curvature in the chemotactic behavior and diminished turning and straight-swimming^11^. Thus, NCX and PMCA seem to cooperate to maintain [Ca^2+^]_i_ in the ascidian sperm. On the other hand, the capacitative Ca^2+^ entry, which is mediated by the store-operated Ca^2+^ channel, seems to mediate flagellar movements to establish the chemotactic behavior of the ascidian sperm^23^. The capacitative Ca^2+^ entry is induced by depletion of internal Ca^2+^ stores and lasts for a few minutes. Here, we propose a new working hypothesis showing the molecular mechanism of sperm chemotaxis in the ascidian *C*. *intestinalis* (Fig. 9). When the spermatozoon is activated by SAAF, PMCA may induce a decrease in [Ca^2+^]_i_, resulting in depletion of internal Ca^2+^ stores and induces capacitative Ca^2+^ entry. When SAAF concentration increases, PMCA may be continuously activated by SAAF, and Ca^2+^ may be kept at low levels, though the capacitative Ca^2+^ entry through the Ca^2+^ channels may occur continuously (ascending phase). When SAAF concentration decreases, SAAF may detach from PMCA and become inactivated, resulting in [Ca^2+^]_i_ bursts by the capacitative Ca^2+^ entry (descending phase). Intracellular Ca^2+^ is excreted by NCX and PMCA. Finally [Ca^2+^]_i_ reaches low levels (refractory phase). It is still unknown whether Orai1 and Stim1, which compose the store-operated Ca^2+^ channels, are involved in the sperm chemotaxis system. On the other hand, CatSper, which is the Ca^2+^ channel having an important role in mammalian sperm, also exists in the ascidian^54^. Function of CatSper in the ascidian sperm is still unknown. CatSper might act as the Ca^2+^ channel in the hypothesis instead of the store-operated Ca^2+^ channel. Furthermore, difference of amino-acid sequences between the two splice variants of PMCA was only found in the C-terminus region (see Supplemental Fig. S2). The C-terminus tail of the mammalian PMCA contains a PDZ-binding domain and it interacts with many other proteins^13^. Because none of the ascidian PMCA variants contain a PDZ-domain, the C-terminus region of the ascidian PMCA may have a role in a novel signaling cascade. Further studies on the roles of PMCA and Ca^2+^ channels will provide new insights into the mechanisms and functions of [Ca^2+^]_i_ changes.

## Materials and Methods

### Materials

The ascidian *C*. *intestinalis* (type A; also called *C*. *robusta*) was obtained from the National BioResource Project for *Ciona* (http://marinebio.nbrp.jp/). Sperm of the ascidian was obtained as described previously^22^. Artificial seawater (ASW) contained 462 mM NaCl, 9 mM KCl, 10 mM CaCl_2_, 48 mM MgCl_2_, and 10 mM HEPES-NaOH (pH 8.2). High and low Ca^2+^-ASW was prepared as the ASW but with 0.01, 0.1, 1, 50 or 100 mM CaCl_2_ and 5 mM EGTA. EGTA, Fluo-4 AM, Cremophor EL and 5(6)-Carboxyeosin diacetate succinimidyl ester (CEDA) were purchased from Thermo Fisher Scientific (Tokyo, Japan); Caloxin 2A1 was purchased from Peptide Institute Inc. (Osaka, Japan); Fluo-8H was purchased from Nacalai Tesque, Inc. (Kyoto, Japan). SAAF and its derivatives were synthesized as described previously^20,55^.

### Preparation of the SAAF-resin

Bio-SAAF was immobilized on streptavidin-coated beads by using the affinity of biotin for streptavidin (TetraLink™ Tetrameric Avidin Resin; Promega Japan, Tokyo, Japan). Binding capacity of the beads was 30 nmol of biotin/mL. In general, 150 nmol of bio-SAAF was added to 10 mL of 50% slurry of streptavidin-coated beads. The beads were previously obtained by washing with 10 mL of PBS three times and incubating for 30 min at room temperature on a rotary mixer at low speed. The unbound material was carefully removed, and the beads were washed with 10 mL of PBS three times and immediately used or stored at 4 °C. For simplicity, the SAAF bound to the beads is referred to as the SAAF-resin and the beads bound with biotin as the control resin.

### Sequence alignment and phylogenetic analysis

Protein sequences of the PMCA family were obtained from the NCBI database (https://www.ncbi.nlm.nih.gov/protein/). Sequence alignments and phylogenetic analysis for the collected sequence data were carried out by GENETYX (Genetyx Co., Tokyo, Japan) with ClustalW (http://www.clustal.org/clustal2/). Accession numbers of the used sequences: Human ATP2B1 isoform 1a [*Homo sapiens*], NP_001001323.1; Human ATP2B2 isoform 1 [*Homo sapiens*], NP_001001331.1; Human ATP2B3 isoform 3b [*Homo sapiens*], NP_001001344.1; Human ATP2B4 isoform 4a [*Homo sapiens*], NP_001001396.1; Mouse Atp2b1 [*Mus musculus*], NP_080758.1; Mouse Atp2b1 isoform 1 [*Mus musculus*], NP_001031761.1; Mouse Atp2b1 isoform 2 [*Mus musculus*], NP_001297466.1; Mouse Atp2b1 isoform a [*Mus musculus*], NP_001161421.1; Rat Atp2B3 [*Rattus norvegicus*], NP_579822.1; Chicken ATP2B1 [*Gallus gallus*], NP_001161474.1; Crow Atp2B1 isoform X1 [*Corvus cornix cornix*], XP_019135366.1; Lizard predicted Atp2b4 isoform X1 [*Anolis carolinensis*], XP_008107875.1; Bearded dragon Atp2b1 isoform X2 [*Pogona vitticeps*], XP_020633124.1; Xenopus predicted ATP2B1 isoform X1 [*Xenopus tropicalis*], XP_017947410.1; Xenopus Atp2b3 [*Xenopus laevis*], NP_001087020.1; Goby Atp2b1 [*Boleophthalmus pectinirostris*], XP_020796624.1; Medaka fish Atp2b1 isoform X1 [*Oryzias latipes*], XP_020569770.1; Zebrafish Atp2b2 [*Danio rerio*], NP_001116710.1; Coelacanth predicted Atp2b3 isoform X1 [*Latimeria chalumnae*], XP_006002568.1; Shark Atp2b1 isoform X1 [*Rhincodon typus*], XP_020391438.1; Amphioxus predicted PMCA2-like isoform X1 [*Branchiostoma belcheri*], XP_019624340.1; Sea Urchin sperm PMCA [*Strongylocentrotus purpuratus*], NP_001028822.1; Drosophila PMCA isoform Q [*Drosophila melanogaster*], NP_001188516.1.

### Preparation of the plasma membrane fraction

Sperm or *Sf*9 cells were lysed in ice-cold PBS containing Complete, EDTA-free (Roche Diagnostics, Tokyo, Japan) using N_2_ cavitation (5.52 MPa, 15 min, on ice). Cell lysates were centrifuged at 13,200 *g* for 10 min at 4 °C to obtain the post-nuclear supernatant. The post-nuclear supernatant was centrifuged at 543,000 *g* for 1 hr at 4 °C to obtain the membrane fractions (pellet). The membrane fractions were resuspended in PBS containing 1% NP-40, sonicated, and then used for SAAF pull-down assay, molecular interaction assays, and for ATPase activity assays.

### SAAF pull-down assay and mass spectrometry

The SAAF-resin was mixed with the membrane fractions at 4 °C for 1 h with rotation, washed with three bed volumes of PBS, and eluted with the elution buffer (8 M Urea, 4% CHAPS, 40 mM Tris-HCl, pH 7.4). The membrane fractions (input), the washes, and the elutes were collected in NuPAGE LDS sample buffer and resolved on NuPAGE SDS–PAGE gel (3–8% Tris-Acetate, Novex, Carlsbad, CA, USA). The resulting gel was stained with SYPRO^®^ Ruby (Thermo Fisher Scientific) according to the manufacturers’ instructions. Protein bands were excised and subjected to for MALDI-TOF/MS as described previously^56^. Briefly, gels were dehydrated and then re-hydrated in 20 ng/μL trypsin or lysyl endopeptidase in 100 mM NH_4_HCO_3_ for 12 h at 37 °C. Peptides were subjected to MALDI-TOF/MS and analyzed by Autoflex II system (Bruker Daltonics K.K., Yokohama, Japan). Proteins were identified by peptide mass fingerprinting (PMF) using MASCOT search with the lab-made protein databases of *C*. *intestinalis*^56^.

### Cloning and protein expression

The cDNAs of *Atp2b* were amplified from RNA, which was isolated from the *C*. *intestinalis* testis by reverse transcription-polymerase chain reaction (RT-PCR) using the following primers: forward, 5′-TAGCGTTACTGCTGCT-3′, and reverse, 5′-TGCCAGCCATGATGTTTTCAGAG-3′. The PCR products were subcloned into the pCR^®^8 TOPO^®^ TA vector (Thermo Fisher Scientific). The construct was controlled by sequencing. Site-directed mutagenesis was carried out to obtain the mutant clone in the appropriate vector. *Atp2b*-var.b was used as the target, and experiments were performed according to the manufacturers’ standard protocol (QuikChange, Stratagene, Cedar, Creek, TX, USA). The following primers were used: *mutation1* (W395A_R396A_K409A) 5′-GGT TTT ACA gcc gcg AGC GAA TGC ACT TCA GTT TAC ATC C-3′ (forward) and 5′-AT AAT GAA GAA cgc GAC AAA ATA TTG GAT GTA AAC TGA AG-3′ (reverse), *mutation2* (R869A_K874A_W880A) 5′-C GCC TGC ATC CTC gcg GAC AGT CCC CTG gcc GCA GTC CAA-3′ (forward) and 5′-C CAT GAT AAG ATT CAC cgc TAA CAT TTG GAC TGC cgc CAG-3′ (reverse), *mutation3* (R951A_H956A_H962A) 5′-GAC ATC GAC AGC GGA gcc GGG CTC AGT CTT gcc TCA GCT C-3′ (forward), and 5′-AA GAC GAT GGT GAA cgc CTG GGT GGG AGC TGA ggc AAG AC-3′ (reverse). The nucleotides that determine the mutation are indicated in lowercase letters. Baculoviruses expressing wild-type and mutant *Atp2b*-var.b were generated using the BaculoDirect Baculovirus Expression System (Thermo Fisher Scientific) according to the manufacturers’ instructions.

### Reverse transcriptase polymerase chain reaction (RT-PCR)

Total RNA was extracted from each tissue using RNeasy Mini Kit (Qiagen, Hilden, Germany) and the concentration of RNA was determined by absorbance at 260 nm in relation to absorbance at 280 nm. One microgram of total RNA was reverse transcribed to cDNA by Transcriptor First Strand cDNA Synthesis Kit (Roche Diagnostics) using anchored-oligo(dT)_18_ primer and random hexamer primer. The 50-µl reaction mixture contained 32 µl nuclease-free water, 1.0 µl cDNA, 1.5 µl (10 µmol/l) each primer, 5 µl (2 mM) dNTPs, 3 µl (25 mM) MgSO_4_, 5 µl (10×) KOD plus ver.2 buffer and 1 µl KOD plus (TOYOBO, Osaka, Japan). The primers were used for detecting the 20^th^ and 22^nd^ exons: 5′-CCC ATC TTT TGC ACA ATC CT-3′ (forward) and 5′-TTC TTC TTC CTC GTC GTC GT-3′ (reverse), for detecting the 20^th^ and 21^st^ exons: 5′-GGC ACG CGT TCT ATC AAC TT-3′ (forward) and 5′-TTT ACT GCA GCA GCA GAT GG-3′ (reverse) were used. For the household gene, glyceraldehyde-3-phosphate dehydrogenase (*Gapdh*): 5′-ACC CAG AAG ACA GTG GAT GG -3′ (forward) and 5′- CAG GAC ACC AGC TTC ACA AA -3′ (reverse). The amplification cycle started with denaturation at 94 °C for 2 min, followed by 30 cycles of denaturation at 98 °C for 10 s, annealing at 55 °C for 30 s, and 68 °C for 1 min. PCR products were analyzed on 2% (wt/vol) agarose gels stained with 0.5 µg/ml ethidium bromide and were visualized under UV light. Images of the gels were captured by using a gel imaging device (Printgraph, AE-6932, ATTO, Tokyo Japan). Acquired images were processed by Adobe Photoshop and Adobe Illustrator (Adobe Systems, San Jose, CA, USA).

### Western blotting and immunostaining

Samples were solubilized directly using SDS-PAGE sample buffer (Laemmli) or the NuPAGE LDS sample buffer (Novex, Carlsbad, CA, USA). In the case of spermatozoa or *Sf*9 cells, these solubilized samples were treated with Benzonase Nuclease (Novagen, Billerica, MA, USA). Proteins were separated by an SDS-PAGE (Laemmli) or by NuPAGE SDS-PAGE Gel System using 3–8% Tris-Acetate Gels (Novex, Carlsbad, CA, USA) and transferred to PVDF membranes. The anti-pan PMCA antibody [5F10], (ab2825; Abcam, Tokyo, Japan), horse radish peroxidase (HRP)-conjugated anti-mouse IgG (GE Healthcare Japan, Tokyo, Japan), and ECL Prime (GE Healthcare Japan) were used to detect the PMCA. To detect *C*. *intestinalis* Atp2b-var.a or Atp2b-var.b, polyclonal antibodies specific to each variant were developed using rabbits (Eurofins Genomics, Tokyo, Japan). The epitope sequences for Atp2b-var.a and Atp2b-var.b were ASEENSTENNQTQNSVA and ARHHSSHHAHLEPV, respectively. For detecting the anti-Atp2b-var.a and Atp2b-var.b antibodies, HRP-conjugated anti-rabbit IgG (Bethyl Laboratories; Montgomery, TX, USA), and ECL Prime (GE) were used. Luminescence was detected using an X-ray film (Fujifilm, Tokyo Japan) and images were acquired using a scanner GT-X770 (Epson, Suwa, Japan) (Fig. 1A,E) or imaged by a gel imager (EzCapture II; ATTO) (Figs 1F and 3). Acquired images were processed by Adobe Photoshop and Adobe Illustrator (Adobe Systems). Specificity of the anti-Atp2b-var.b was shown in Supplemental Fig. S7.

For immunostaining, the sperm suspension was put onto a coverslip and fixed with 4% paraformaldehyde for 10 min, after which it was washed twice with ASW for 5 min. The fixed sperm was permeabilized with 0.1% NP-40 for 15 min, blocked with 1% BSA for 1 h and incubated with 5 µg/mL 5F10 or x1/500 anti-Atp2b-var.b antibody with 1% BSA in PBS for 45 min. The sperm was washed 3 times with PB and incubated with x1/2000 Alexa 488-conjugated anti-mouse IgG(H + L) (ThermoThermo Fisher Scientific) or x1/2000 DyLight550-conjugated anti-rabbit IgG (Abcam) for 30 min, respectively. After washing two times with PBS, the sperm was observed using a fluorescent microscope (IX-71; Olympus). Images were recorded on a PC connected to a digital CCD camera (Retiga Exi; QImaging, Surrey, Canada) using an imaging application (TI workbench)^57^. Acquired data was processed by Adobe Photoshop and Adobe Illustrator (Adobe Systems).

### Molecular interaction assay

Interaction between SAAF and PMCA was investigated by a highly sensitive 30-MHz quartz crystal microbalance (QCM; NAPiCOS; Nihon Dempa Kogyo, Tokyo, Japan). The first channel of a sensor chip was coated with PMCA-expressing *Sf*9 membranes (5 mg/mL of proteins) suspended in PBS (pH 7.4) containing 0.1% of NP-40. The second channel of the same sensor chip was coated with *Sf*9 membranes (5 mg/mL of proteins), as a reference. The sensor chip was washed three times with PBS, placed into the chamber, perfused with PBS until the frequency was stabilized. Then, the sensor chip was perfused with SAAF (125 µM) dissolved in PBS, and the change in frequency was recorded. In order to examine SAAF and mutant PMCA interactions, the first channel of a sensor chip was coated with mutant PMCA-expressing *Sf*9 membranes. The second channel on the same sensor chip was coated with PMCA-expressing *Sf*9 membranes, as a reference. After washing, perfusion, and blocking the sensor chip, it was perfused with SAAF, and the change in frequency was recorded. All experiments were performed at 20 °C, with a flow rate of 50 μL/min. To evaluate the binding affinity, changes in the frequency of cumulative perfusion were analyzed. Affinities were evaluated by the Michaelis-Menten equation, and fitted curves were obtained by calculating the average of three experimental values. The dissociation constant (K_D_) was calculated with NAPiCOS Analysis software (Nihon Dempa Kogyo).

### Assay for ATPase activity of PMCA

Enzyme activity of PMCA from *Ciona* sperm and *Sf*9 membranes was determined by measuring the inorganic phosphate produced using a coupled enzyme assay kit (EnzChek® Phosphate Assay Kit, Thermo Fisher Scientific) according to the manufacturers’ instructions. In brief, membranes were incubated for 5 min at 25 °C in a reaction mixture containing 50 mM Tris-HCl, pH 7.4, 1 mM MgCl_2_, 0.2 mM 2-Amino-6-mercapto-7-methylpurine riboside (MESG) as a substrate, 1 U/mL purine nucleoside phosphorylase (PNP) as a converting enzyme, and 100 µM CaCl_2_. The enzymatic reaction was initiated by addition of 1 mM ATP. ATPase activity was calculated based on the difference in ATP hydrolysis between samples incubated in the presence and absence of 10 µM SAAF or bio-SAAF. Enzymatic conversion of MESG by PNP was measured for 5 min at room temperature at a wavelength of 360 nm in a recording spectrophotometer (Ultrospec2100pro, Amersham Biosciences).

### Analysis of sperm chemotaxis

Sperm chemotaxis was examined as described previously^24^. Briefly, semen was diluted 10^4^ to 10^5^ times in the medium for experiment (e.g. ASW containing CEDA or Caloxin 2A1; high and low Ca^2+^-ASW). Theophylline (Sigma-Aldrich Japan, Tokyo, Japan) was added to the suspension in a final concentration of 1 mM for the induction of motility activation which is mediated by an increase in intracellular cAMP^3^, except in a series of Caloxin 2A1 experiments. Series of Caloxin 2A1 experiments including control were performed with the sperm suspension not containing theophylline, since the Caloxin 2A1-treated theophylline-activated sperm completely adhered to the glass slide and there was no free-swimming sperm. The activated-sperm suspension was placed in the observation chamber, and sperm movement around the micropipette tip containing SAAF was recorded. The position of the sperm head was analyzed with Bohboh software (BohbohSoft, Tokyo, Japan)^58^. The parameters of chemotactic activity (trajectory, the distance between the capillary tip and the sperm, and LECI) were calculated as described previously^19^.

### Imaging analysis of [Ca^2+^]_i_ in the flagella of swimming sperm

For Ca^2+^ imaging, Fluo-8H AM (Fig. 7) or Fluo-4 AM (Fig. 8) was used as a fluorescent probe. The dye-loaded sperm was prepared as described previously^24^. Fluorescent images of the sperm were observed by a microscope (IX71, Olympus, Tokyo, Japan), and captured on a PC connected to a digital CCD camera (ImagEM, C9100-13; Hamamatsu Photonics, Hamamatsu, Japan) at 32.5 frames/sec using Aquacosmos (Hamamatsu Photonics), or a digital CCD camera (Retiga Exi; QImaging) at 50 frames/sec using an imaging application (TI workbench^57^), as described previously^24,59^. For fluorescence illumination, a stroboscopic lighting system with a power LED was used as described^24^. Fluorescent signal intensity and sperm flagellar bending were also analyzed using the Bohboh software^58^.

### Statistical analysis

All experiments were repeated at least three times with different specimens. Data is expressed as the mean ± SD. Statistical significance against control (Figs 4B, 5B, 6B and 7), or the normal SW (Fig. 8B) was calculated using the Student’s *t*-test; P < 0.05 was considered significant.

## Electronic supplementary material

Supplemental Figures and Table
