# Supplementary material for: Ca2+ efflux via plasma membrane Ca2+-ATPase mediates chemotaxis in ascidian sperm
Source: Sci Rep. 2018 Nov 9;8:16622. doi: 10.1038/s41598-018-35013-2 (PMC6226504; doi:10.1038/s41598-018-35013-2)
Supplement: Supplementary file 1 — Supplemental Figures and Table [file 41598_2018_35013_MOESM1_ESM.pdf]

Title:

Ca^{2+} efflux via plasma membrane Ca^{2+}-ATPase mediates chemotaxis in ascidian sperm

Authors / Affiliations:

Kaoru Yoshida[1], Kogiku Shiba[2,3], Ayako Sakamoto[2,4], Junpei Ikenaga[2], Shigeru Matsunaga[2,5], Kazuo Inaba[3] and Manabu Yoshida[2]*

[1]*Faculty of Biomedical Engineering, Toin University of Yokohama, Yokohama, Kanagawa 225-8503, Japan*

[2] *Misaki Marine Biological Station, School of Science, the University of Tokyo, Miura, Kanagawa 238-0225, Japan*

[3] *Shimoda Marine Research Center, University of Tsukuba, Shimoda 415-0025, Japan*

Supplemental Information

Supplemental Fig. S1. Identification of SAAF-binding proteins

(A) Molecular structure of bio-SAAF. (B) SAAF-binding proteins were pulled down by a SAAF-immobilized resin. Bio-SAAF was immobilized in a streptavidin-resin. The solubilized membrane fraction of N_2-cavitated *Ciona* sperm with 1% NP-40/PBS, was incubated with the SAAF-resin. SAAF-binding proteins were eluted by 8 M Urea buffer. All these fractions were separated by 4–20 % SDS-PAGE and stained with CBBR. (C) The result of PMF methods was obtained using Mascot search. Three bands (370 kDa, 330 kDa, and 130 kDa) were identified and digested by lysyl endopeptidase (Lys-C) or by trypsin. Digested fragments were measured by MALDI-TOF/MS. Although the Mascot score was low, the top hit among these samples (370 kDa and 330 kDa) showed the same protein ID, a product of the predicted gene model KH.C8.156. The predicted amino acid sequence was subjected to BLAST and it revealed that the protein was similar to ATP2B3 (*Homo sapiens*).

Supplemental Fig. S2. Amino acid sequence alignments of *Ciona intestinalis* Atp2b-var.a [LC271262], Atp2b-var.b [LC271262], and human ATP2B1–4

Supplemental Fig. S3. Genome DNA sequence around the exon 17, 18, and 19 of *Atp2b* locating in chromosome 8. Sequenced data was shown as Genome-Gap seq [LC411940]. Since there is a gap in the sequence between exon 17 and 19 in *C. intestinalis* genome database (Ghost: http://ghost.zool.kyoto-u.ac.jp/cgi-bin/gb2/gbrowse/kh/), KH.C8.156 did not contain exon 18, and predicted exon 19 was modified (yellow), resulting in shift of reading frame. Exon 17, 18, and 19 are shown as blue frames. KhC8 is the contig sequence of chromosome 8 in the genome database.

Methods: Genome DNA was extracted from the *C. intestinalis* sperm by Puregene Cell Kit (Qiagen; Tokyo, Japan). The gap region between exon 17 and 19 was isolated by polymerase chain reaction (PCR) with TaKaRa Ex Taq® (Takara Bio, Kusatsu, Japan) using the following primers: forward, 5′-GAAGAA TATCCTCGGGCACGCGTTCT-3′, and reverse, 5′-GACGATTTGTCCCCACACGAGCTCAA-3′. The PCR products were subcloned into the pGEM®-T Easy vector (Promega; Madison, WI, USA), and sequencing of the construct was outsourced to Fasmac (Atsugi, Japan). The Primers for sequences are 5′-ACTTGGTGGCTACAGACTGT-3′, 5′-AGCCAAGATGTGTGATTCGT-3′, and M13 Forward and Reverse Sequencing Primers.

Supplemental Fig. S4. Full-length gels of Figure 1D.

Supplemental Fig. S5. Full-length gels of Figure 1E.

Supplemental Fig. S6. Full-length gels of Figure 1F.

Supplemental Fig. S7. Absorption experiment of anti-Atp2b_ver.b antibody by antigen peptide. Sperm membrane proteins were separated by NuPAGE SDS-PAGE Gel System using 4–20% Bis-Tris Gels (Novex, Carlsbad, CA, USA) and transferred to PVDF membranes. The anti-pan PMCA antibody [5F10], (ab2825; Abcam, Tokyo Japan) or Atp2b-var.b, polyclonal antibody was used for the detection of PMCA. Before incubation with blotted PVDF membrane, these antibodies were incubated with or without the antigen peptide. The band of PMCA was disappeared only using Atp2b-var.b, polyclonal antibody absorbed with the antigen peptide.

Supplemental Fig. S8. Effect of Caloxin 2A1 on frequency distribution of $[Ca^{2+}]_i$ in the sperm heads. (A) Baseline of the $[Ca^{2+}]_i$ represented by minimum $[Ca^{2+}]_i$ in the sperm head around the tip of a capillary containing 1 μM SAAF. We conclude that the baseline of the $[Ca^{2+}]_i$ of the sperm showing sperm chemotactic behavior ranges from 0.5 to 1.5. (B) Maximum $[Ca^{2+}]_i$ around the tip of a capillary containing 1 μM SAAF. Data of (B) is the same as shown in Fig. 7B. The sperm were observed in ASW (control) or treated with 1 or 2 mM Caloxin 2A1. $[Ca^{2+}]_i$ was expressed as F/F_0 as shown in Fig 7.

Supplemental Fig. S9. Effects of extracellular Ca^{2+} on Ca^{2+} bursts on chemotactic behavior of sperm. (A) Definition of the swimming episodes in SAAF concentration gradient and the angular direction of the sperm swimming (θ) relative to the attractant source (the tip of the capillary, O). Swimming episodes are arbitrarily divided by θ into 4 phases: ascending (yellow), proximal (green), descending (blue), and

distal (pink). The θ value at position P is calculated from the 2 vectors OP (blue arrow) and P1P2 (orange arrow), where P1 and P2 are the positions at 2 frames before and after P, respectively. Modified from Shiba et al., 2008. (B-C) Frequency distribution of θ at the Ca^{2+} burst initiation point during sperm chemotaxis for the micropipette tip containing 1-μM SAAF in normal (B, 10 mM) and high-Ca^{2+}-ASW (C, 50 mM and 100 mM). N=91 (modified from Shiba et al., 2008.), 28 and 43 in 10 mM, 50 mM and 100 mM Ca^{2+}-ASW respectively. Method of the analysis is shown in Shiba et al. (2008).

References

Shiba, K., Baba, S. A., Inoue, T. & Yoshida, M. Ca^{2+} bursts occur around a local minimal concentration of attractant and trigger sperm chemotactic response. *Proc Natl Acad Sci USA* **105**, 19312-19317 (2008).

A

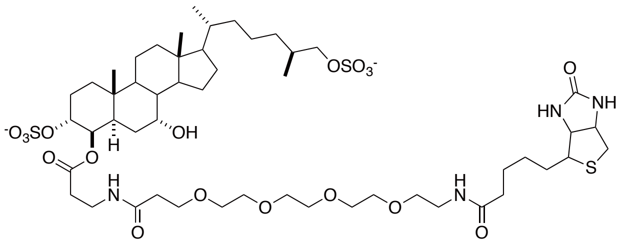

B

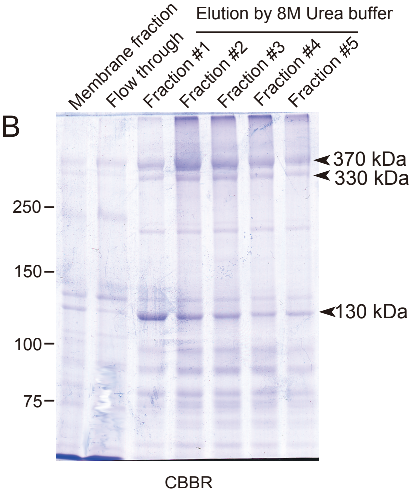

C

Sample Protein	Digestion	Mascot score	Protein ID	Predicted MW(kDa)	BLAST acc.#	Remarks
370 kDa	Lys-C	42	KH.C8.156.v1.B.SL1-1	107	NM_021949	ATP2B3
370 kDa	Trypsin	58	KH.C8.156.v1.B.SL1-1	107	NM_021949	ATP2B3
330 kDa	Lys-C	42	KH.C8.156.v1.B.SL1-1	107	NM_021949	ATP2B3

Fig S1. Yoshida et al.

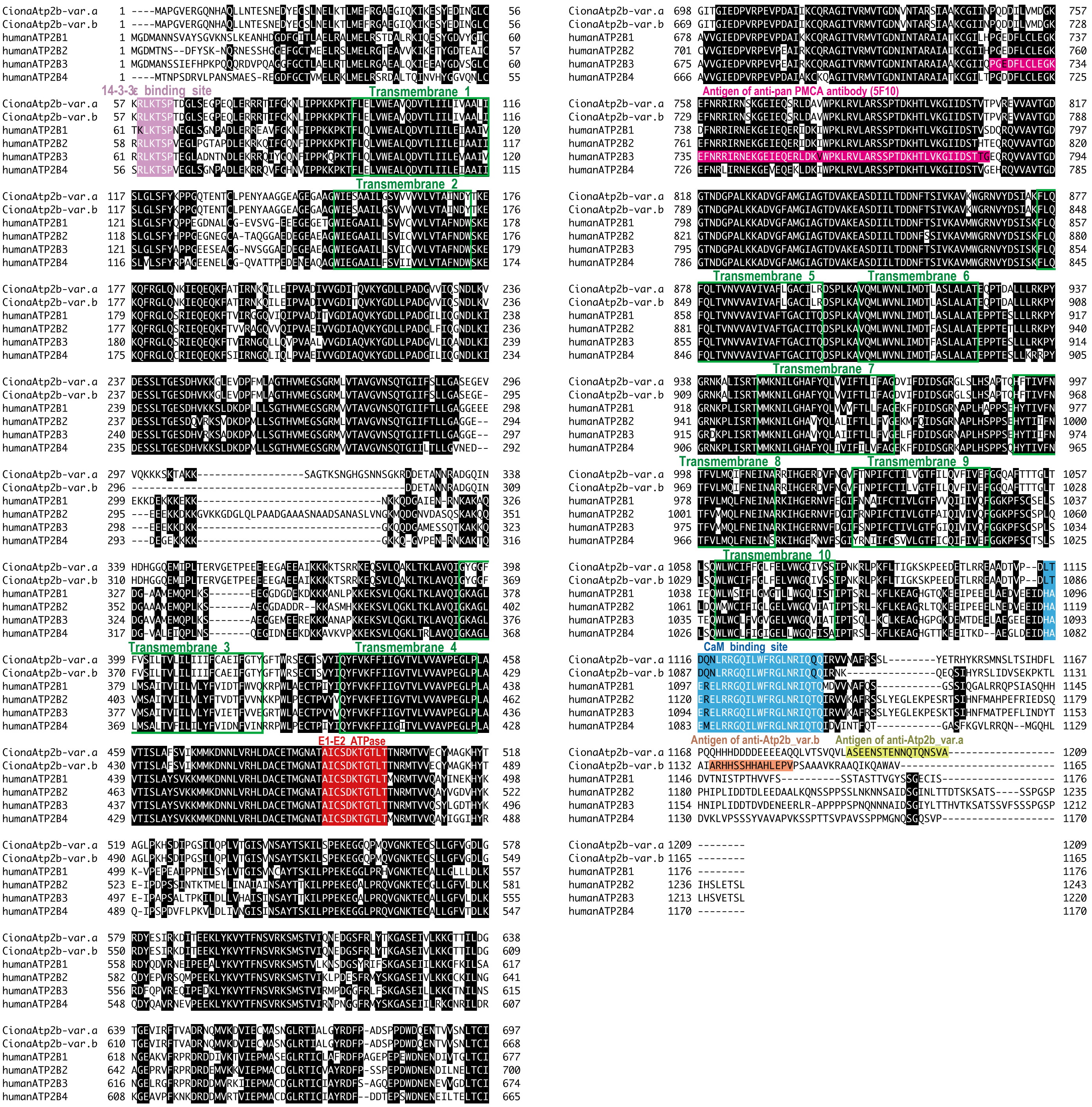

Fig S2. Yoshida et al.

```
         Contig  11281 :TAAAAAAGTCTGGATTTTTGCAAAATGTACAATGCAAAAAATTTATGGCAAATAAAAATA
          KhC8 2482938 :TAAAAAAGTCTGGATTTTTGCAAAATGTACAATGCAAAAAATTTATGGCAAATAAAAATA

         Contig  11341 :TTCACTTTTTGGCAGATTTTTGCAAAAATATGCAATTACAAATCAAGCAATAATTACACT
          KhC8 2482998 :TTCACTTTTTGGCAGATTTTTGCAAAAATATGCAATTACAAATCAAGCAATAATTACACT

                                                                Exon 17
         Contig  11401 :ACTTAATTTTCTAATCCACTCCTCCCAAATACAGGACAGTCCCCTGAAAGCAGTCCAAAT
          KhC8 2483058 :ACTTAATTTTCTAATCCACTCCTCCCAAATACAGGACAGTCCCCTGAAAGCAGTCCAAAT

         Contig  11461 :GTTATGGGTGAATCTTATCATGGACACTCTCGCATCACTTGCTCTTGCTACGGAGCAACC
          KhC8 2483118 :GTTATGGGTGAATCTTATCATGGACACTCTCGCATCACTTGCTCTTGCTACGGAGCAACC

         Contig  11521 :TACGGATGCTCTTCTGCTCCGGAAGCCCTACGGCCGCAACAAGGCCCTTATTTCACGAAC
          KhC8 2483178 :TACGGATGCTCTTCTGCTCCGGAAGCCCTACGGCCGCAACAAGGCCCTTATTTCACGAAC

         Contig  11581 :AATGATGAAGAATATCCTCGGGCACGCGTTCTATCAACTTGTGGTCATCTTCACGCTTAT
          KhC8 2483238 :AATGATGAAGAATATCCTCGGGCACGCGTTCTATCAACTTGTGGTCATCTTCACGCTTAT
Genome-Gap seq       1 :      GAAGAATATCCTCGGGCACGCGTTCTATCAACTTGTGGTCATCTTCACGCTTAT

         Contig  11641 :CTTTGCTGGCAAGTTGCTAGTTTTTAAACCTACTTCAGATTTTTTARAATAGGTTTATTA
          KhC8 2483298 :CTTTGCTGGCAAGTTGCTAGTTTTTAAACCTACTTCAGA-TTTTTAGAATAGGTTTATTA
Genome-Gap seq      55 :CTTTGCTGGCAAGTTGCTAGTTTTTAAACATACTTCAGATTTTTTAAAATAGGTTTATT-

         Contig  11701 :TTTTTATAAGAAATAGTACTGATATTTGTAAAATATAGGTGAAAAAGGGGAAAAATACAT
          KhC8 2483358 :TTTTTATACGACAT
Genome-Gap seq     115 :TTTTTATAAGAAATAGTACTGATATTTGTAAAATATAGGTGAAAAAGGGGAAAAATACAT

         Contig  11761 :GTAAATGTGGCAGAAATCCTGACATAAAAAATACACTATTTGTAAACGAATCACACATCT
Genome-Gap seq     175 :GTAAATGTGGCAGAAATCCTGACATAAAAAATACACTATTTGTAAACGAATCACACATCT

         Contig  11821 :TGGCTATTTTATTTTTTAAGAGAGGAAAAAAAACAATTCGCGATTGCCTACAATAATTTA
Genome-Gap seq     235 :TGGCTATTTTATTTTTTAAGAGAGGAAAAAAAACAATTCGCGATTGCCTACAATAATTTA

         Contig  11881 :TGTAACTATTTTAAAGTTATCAATTTTGTATCTACCCTTATCTACTACTCATGAAGTTGG
Genome-Gap seq     295 :TGTAACTATTTTAAAGTTATCAATTTTGTATCTACCCTTATCTACTACTCATGAAGTTGG

         Contig  11941 :ACAATTTAACTAAATAACATTCAAGATCAAGAGCCACGAAAAAATACGTAGCAGAAAATC
Genome-Gap seq     355 :ACAATTTAACTAAATAACATTCAAGATCAAGAGCCACGAAAAAATACGTAGCAGAAAATC

                                                                      Exon 18
         Contig  12001 :AAGTCTATCAAGTCAAACTATTTGTATTTAATTTCCAGGTGACGTCATCTTTGACATCGA
Genome-Gap seq     415 :AAGTCTATCAAGTCAAACTATTTGTATTTAATTTCCAGGTGACGTCATCTTTGACATCGA

         Contig  12061 :CAGCGGACGCGGGCTCAGTCTTCACTCAGCTCCCACCCAGCATTTCACCATCGTCTTCAA
Genome-Gap seq     475 :CAGCGGACGCGGGCTCAGTCTTCACTCAGCTCCCACCCAGCATTTCACCATCGTCTTCAA

         Contig  12121 :CACCTTTGTACTGATGCAGATCTTCAATGAAATAAATGCACGGAGGATTCACGGGGAGAG
Genome-Gap seq     535 :CACCTTTGTACTGATGCAGATCTTCAATGAAATAAATGCACGGAGGATTCACGGGGAGAG

         Contig  12181 :GGATGTCTTCAATGGAGTCTTCACCAACCCCATCTTTTGCACAATCCTCGTCGGGACTTT
Genome-Gap seq     595 :GGATGTCTTCAATGGAGTCTTCACCAACCCCATCTTTTGCACAATCCTCGTCGGGACTTT
```

Fig. S3 Yoshida et al.

```
       Contig  12241 :TATCCTCCAGGTTTGGAAGTTATGGATAATTCTTATTTATTTTTGATATGAGTGAGGTCC
Genome-Gap seq   655 :TATCCTCCAGGTTTGGAAGTTATGGATAATTCTTATTTATTTTTGATATGAGTGAGGTCC

       Contig  12301 :TTATTTCATATGAGTGAAGTGAGGTCCTTATTTCATATGAGTGAAGTGAGGTCCTTATTT
Genome-Gap seq   715 :TTATTTCATATGAGTGAAGTGAGGTCCTTATTTCATATGAGTGAAGTGAGGTCCTTATTT

       Contig  12361 :CATATGGGTGAAGTGAGGTCCTTATTTCATATGAGTGAAGTGAGGTCCTTATTTCATATG
Genome-Gap seq   775 :CATATGGGTGAAGTGAGGTCCTTATTTCATATGAGTGAAGTGAGGTCCTTATTTCATATG

       Contig  12421 :GGTGAAGTGAGGTCCTTATTGAAATTCACGGCGGTTTGGAAATTGTCTTGAATTTTTCCC
Genome-Gap seq   835 :GGTGAAGTGAGGTCCTTATTGAAATTCACGGCGGTTTGGAAATTGTCTTGAATTTTTCCC

       Contig  12481 :TGAAAAGTTGACTAGTAAGACGCCATGATGGCACAACTGTATACCATTTATGTAAACAGT
Genome-Gap seq   895 :TGAAAAGTTGACTAGTAAGACGCCATGATGGCACAACTGTATACCATTTATGTAAACAGT

       Contig  12541 :CTGTAGCCACCAAGTTTTACTTTATATGGCATTTTTGTGCCACAAAATTTTATTATATAT
Genome-Gap seq   956 :CTGTAGCCACCAAGTTTTACTTTATATGGCATTTTTGTGCCACAAAATTTTATTATATAT

       Contig  12601 :TTAACAAATTAAACTAATGTTATAAAACTGTAAACACATACAGTGAAAAAGGTACTAATA
Genome-Gap seq  1015 :TTAACAAATTAAACTAATGTTATAAAACTGTAAACACATACAGTGAAAAAGGTACTAATA

       Contig  12661 :TTAATACTTTTTTGAAACACAACCTTAAACAAACCCAAAAAAAATGTAAAAAATATGTAT
Genome-Gap seq  1075 :TTAATACTTTTTTGAAACACAACCTTAAACAAACCCAAAAAAAATGTAAAAAA-ATGTAT
     KhC8 2487096 :                TCGAGTCGACTGCAAGTAGTCATCAAAATGTAAAAAATATGTAT

       Contig  12721 :TTGTTTGGTATRGTCCATACATTTCCCCCAAAAAATGAATTCACTCAATTTTAAAATGCG
Genome-Gap seq  1135 :TTGTTTGGTATGGTCCATACATTTCCCCCAAAAAATGAATTCACTCAATTTTAAAATGCG
     KhC8 2487156 :TTGTTTGGTATAGTCCATACATTTCCCCCAAAAAATGAATTCACTCAATTTTAAAATGC-
                                                                       Exon 19
       Contig  12781 :TTTTGTGTTTTGTRTACYGTTTACTTGGTTTATAACATCGATTTCCTCCACTAAGGTTTT
Genome-Gap seq  1195 :TTTTGTGTTTTGTGTACTGTTTACTTGGTTTATAACATCGATTTCCTCCACTAAGGTTTT
     KhC8 2487216 :------GTTTTGTATACCGTTTACTTGGTTTATAACATCGATTTCCTCCACTAAGGTTTT

       Contig  12841 :CATTGTTGAGTTTGGAGGTCAAGCTTTCACCACCACGGGCCTCACCCTCTCACAGTGGCT
Genome-Gap seq  1255 :CATTGTTGAGTTTGGAGGTCAAGCTTTCACCACCACAGGCCTCACCCTCTCACAGTGGCT
     KhC8 2487276 :CATTGTTGAGTTTGGAGGTCAAGCTTTCACCACCACGGGCCTCACCCTCTCACAGTGGCT

       Contig  12901 :TTGGTGCATTTTCTTCGGTTTGTTTGAGCTCGTGTGGGGACAAATCGTCTCCAGCATTCC
Genome-Gap seq  1315 :TTGGTGCATTTTCTTCGGTTTGTTTGAGCTCGTGTGGGGACAAATCGTC
     KhC8 2487336 :TTGGTGCATTTTCTTCGGTTTGTTTGAGCTCGTGTGGGGACAAATCGTCTCCAGCATTCC

       Contig  12961 :TAACAAGAGGCTTCCCAAGTTCTTGACGGTAAAAAAAAACATAAAAATACATTTTTAATA
     KhC8 2487396 :TAACAAGAGGCTTCCCAAGTTCTTGACGGTAAAAAAAAACATAAAAATACATTTTTAATA

       Contig  13021 :TATTTTTGTAATATTTTTGACTACATTGTAATTTTTTTTGTGTTTTTAAAATGTTTGTGA
     KhC8 2487456 :TATTTTTGTAATATTTTTGACTACATTGTAATTTTTTTTGTGTTTTTAAAATGTTTGTGA

       Contig  13081 :AGATTTTGTAAATTAAAAATTGGGATGAAAAATATTCTAAGTAACTGATTGATTTAGTGC
     KhC8 2487516 :AGATTTTGTAAATTAAAAATTGGGATGAAAAATATTCTAAGTAACTGATTGATTTAGTGC
```

Fig. S3 Yoshida et al.

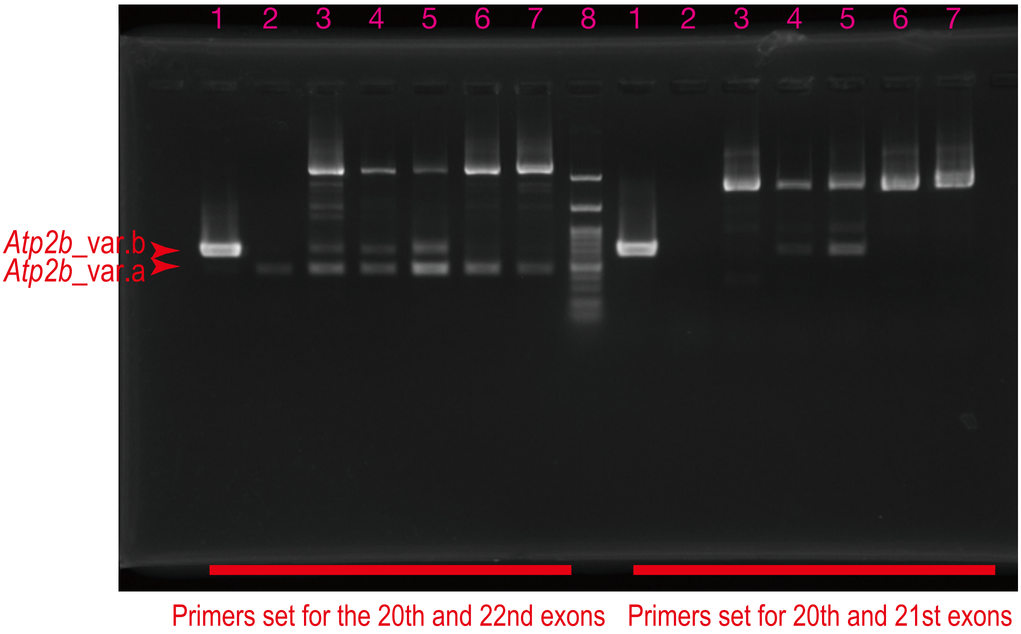

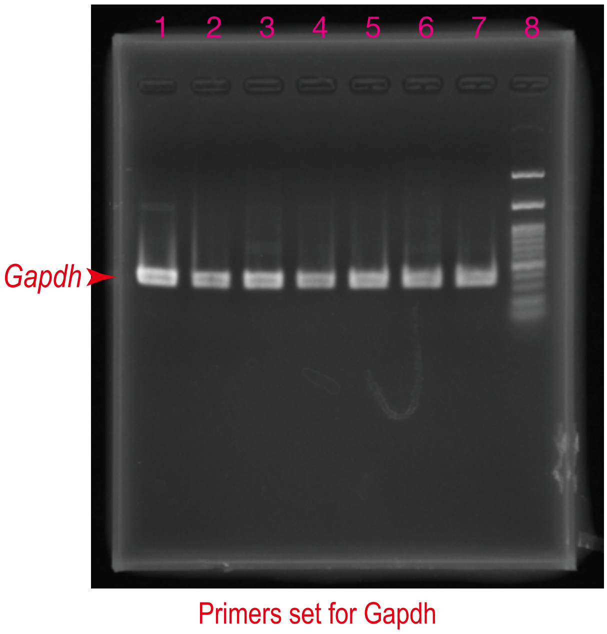

1. Testis
2.Ovary
3. Heart
4. Intestine
5. Stomach
6.Gill
7.Muscle
8. Size Marker

Fig S4. Yoshida et al.

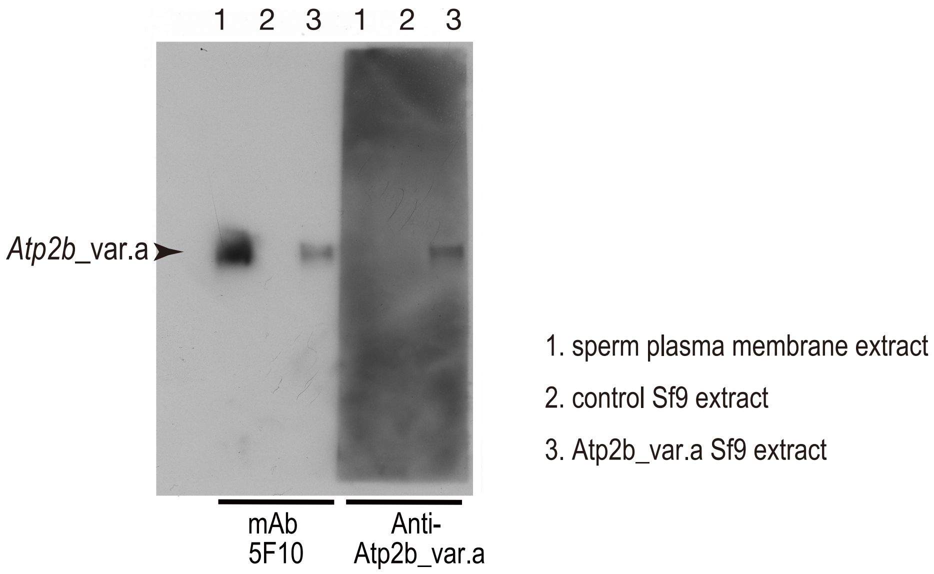

Fig S5. Yoshida et al.

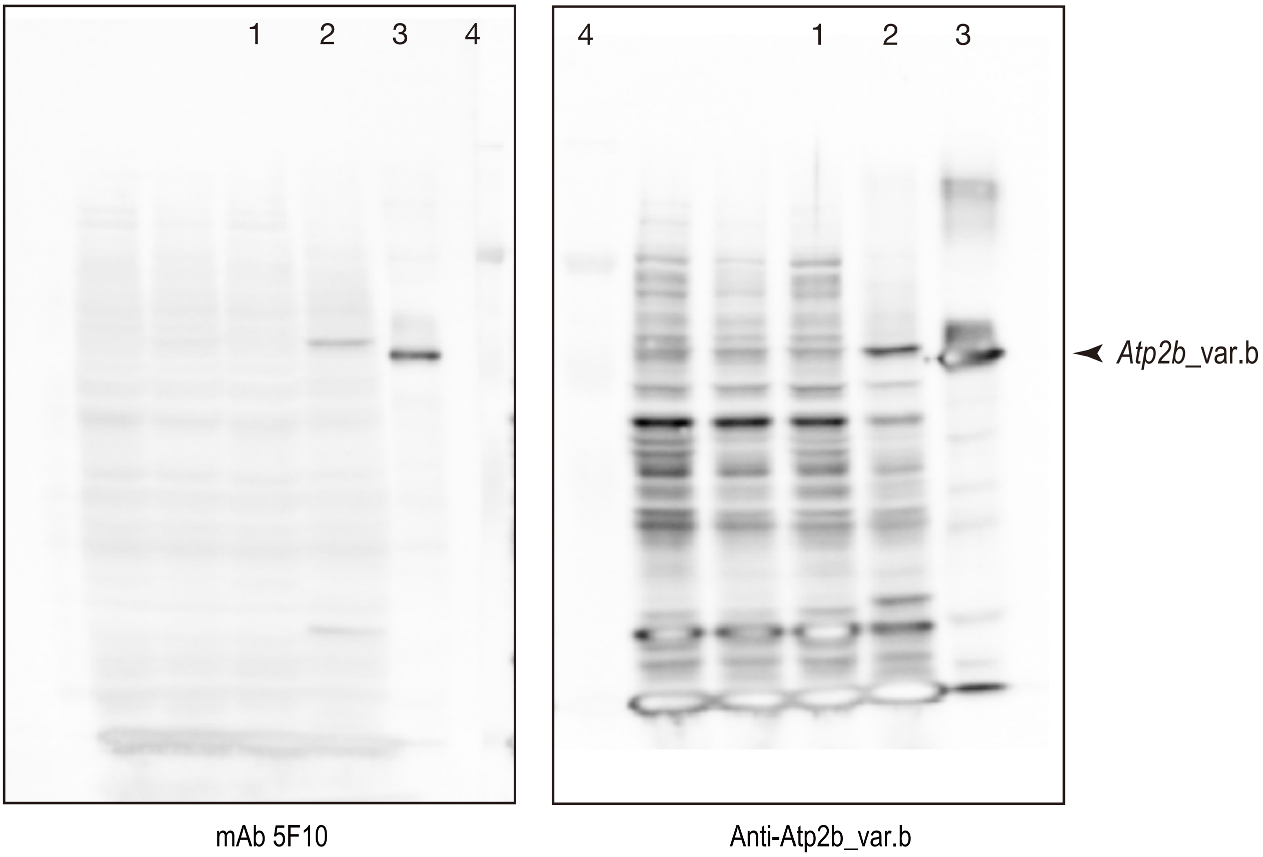

1. control Sf9 extract
2. Atp2b_var.b Sf9 extract
3. sperm plasma membrane extract
4. Molecular weight marker

Fig S6. Yoshida et al.

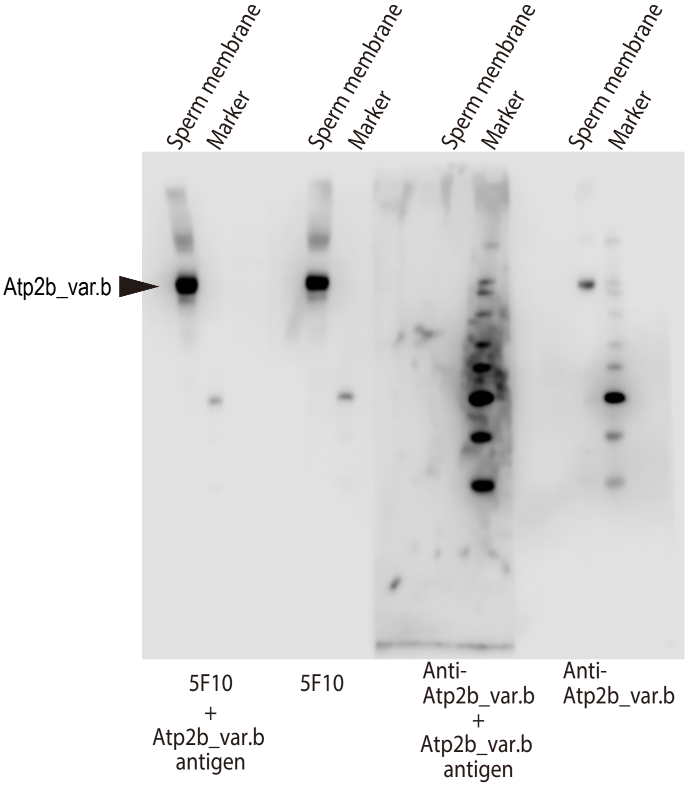

Fig S7. Yoshida et al.

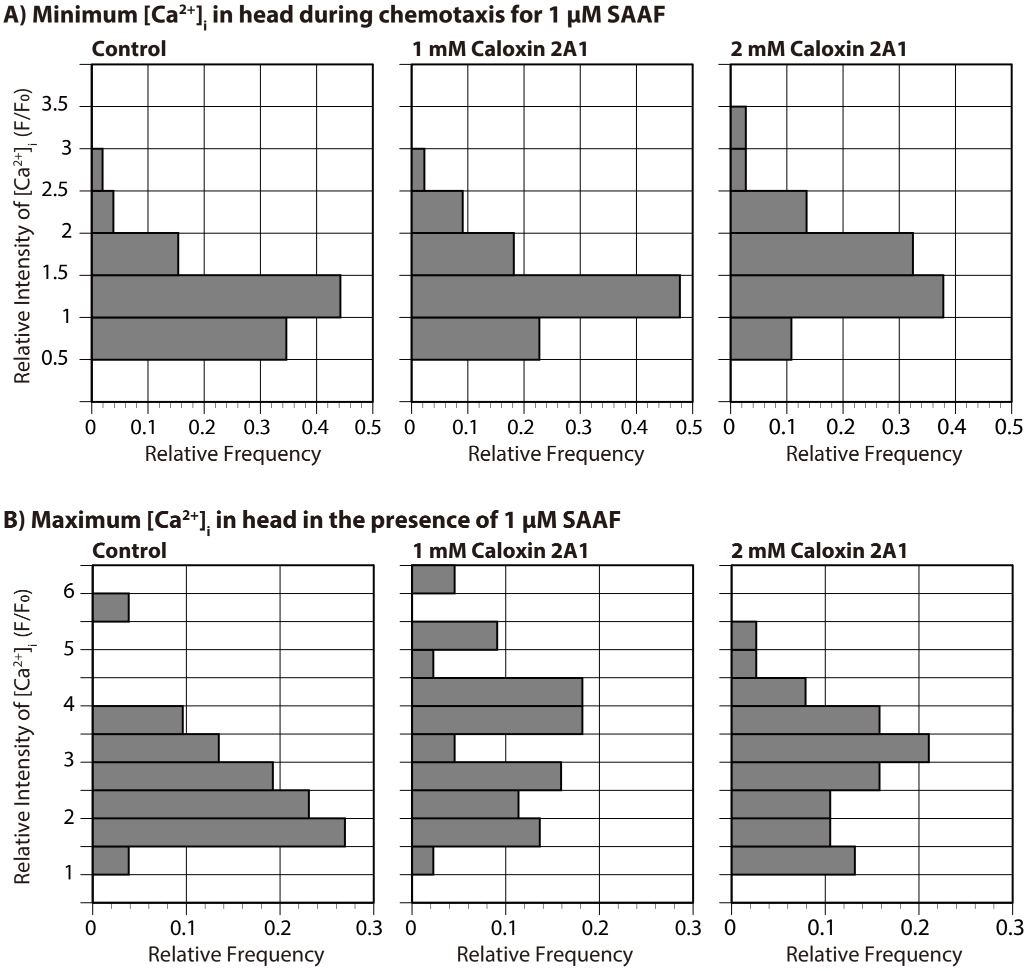

Fig S8. Yoshida et al.

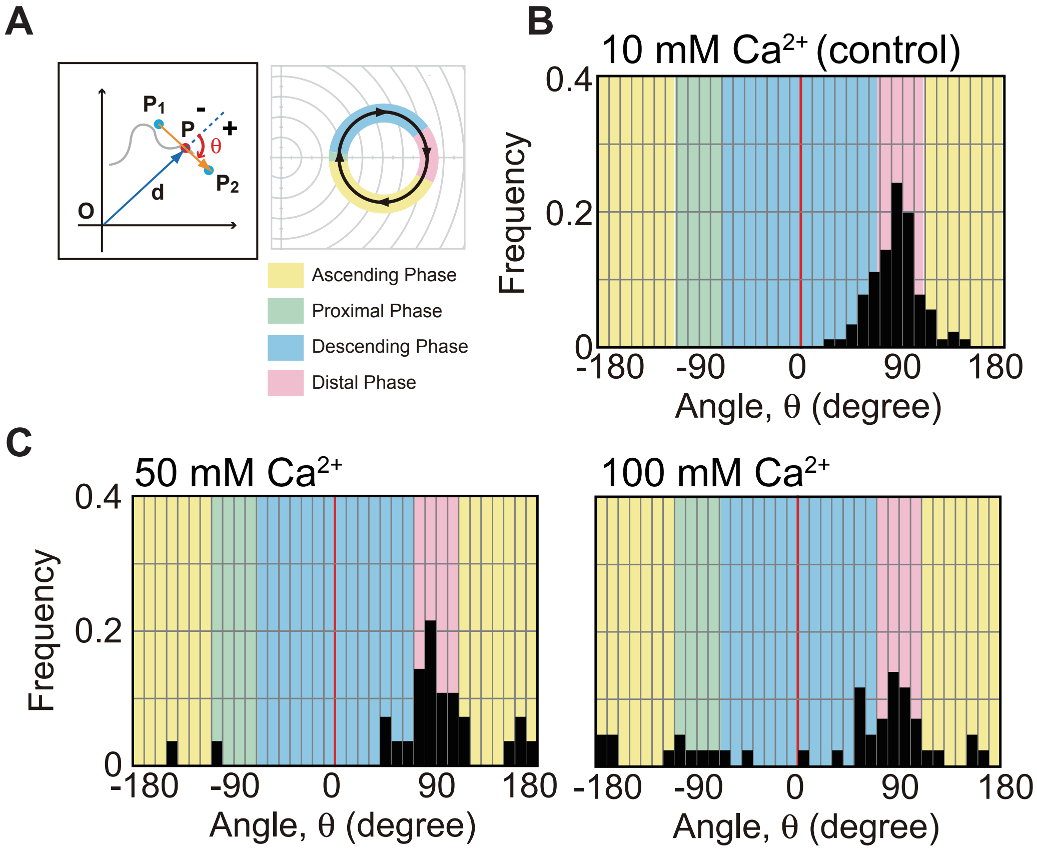

Fig S9. Yoshida et al.

Supplemental Table S1. Effects of extracellular Ca^{2+} on the parameters in SAAF-induced Ca^{2+} bursts

	Duration of Ca^{2+} burst (sec)	$[Ca^{2+}]_i$ peak (F/F_0)	Increasing rate ($F/F_0\ s^{-1}$)	Decreasing rate ($F/F_0\ s^{-1}$)
10 mM Ca^{2+}-ASW	0.42±0.14	1.02±0.24	6.33±3.23	3.49±1.11
50 mM Ca^{2+}-ASW	0.50±0.12	1.35±0.52*	7.89±3.72	3.23±1.21
100 mM Ca^{2+}-ASW	0.63±0.22**	1.32±0.30*	7.98±2.74	2.34±0.79**

Values are expressed as mean ± S.D. (N=14, 18, and 12 in 10 mM, 50 mM, and 100 mM Ca^{2+}-ASW, respectively). Statistical significance with *$P < 0.05$, **$P < 0.01$, or ***$P < 0.001$ (Student's t-test) as compared with 10 mM Ca^{2+}-ASW.
